# Supplementary material for: Infectious Diseases Simulation for Medical Students: Experiential Instruction on Personal Protective Equipment
Source: MedEdPORTAL. 2020 Nov 24;16:11031. doi: 10.15766/mep_2374-8265.11031 (PMC7703477; doi:10.15766/mep_2374-8265.11031)
Supplement: Supplementary file 1 — Prework Slides.pptxSimulation Case 1.docxSimulation Case 2.docxSimulation Case 3.docxExam Questions.docxEvaluation Questions.docx [file mep_2374-8265.11031-s001.zip › A. Prework Slides.pptx]

## Slide 1
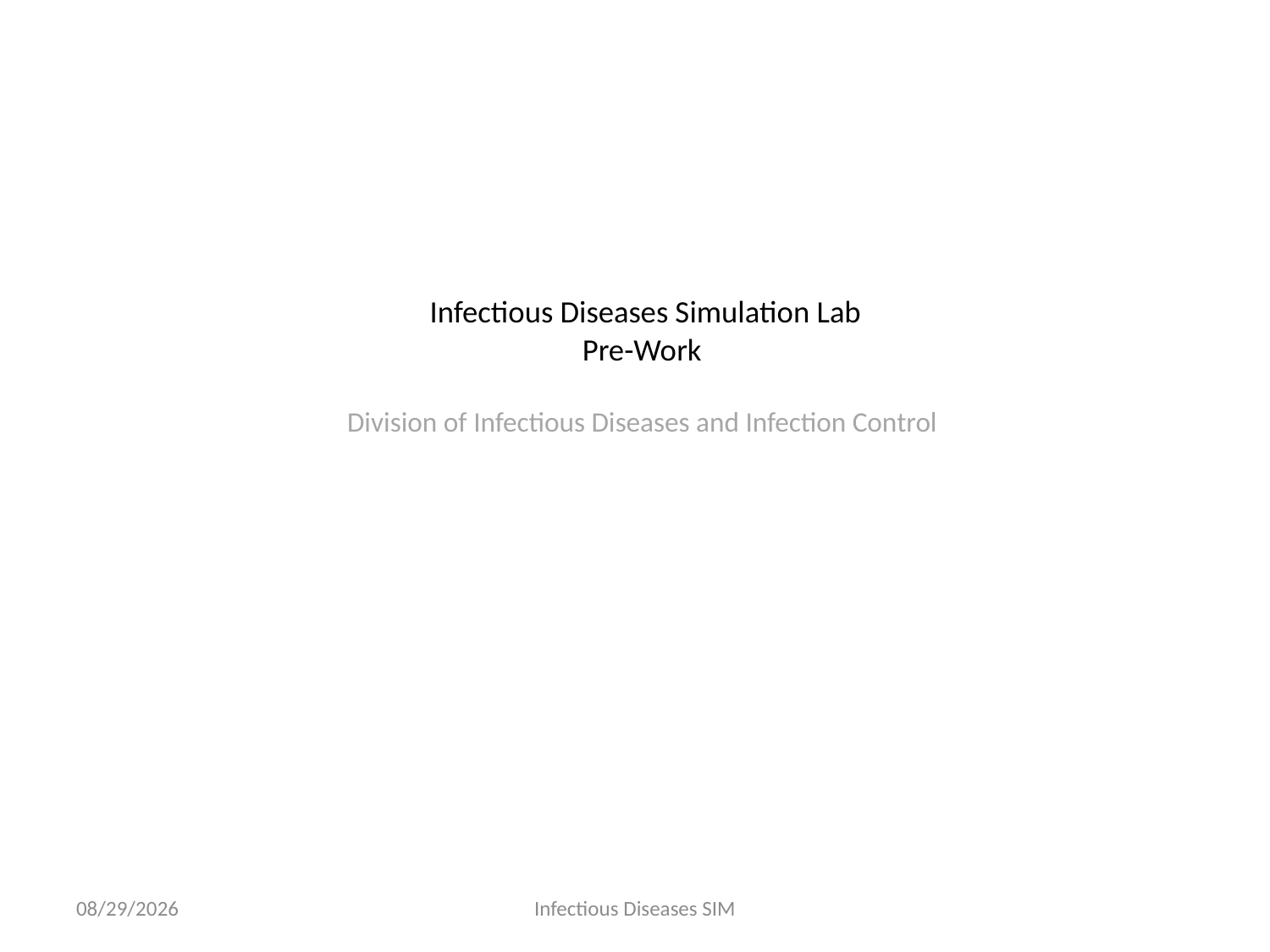

# Infectious Diseases Simulation Lab Pre-Work Division of Infectious Diseases and Infection Control
4/7/2020
Infectious Diseases SIM

## Slide 2
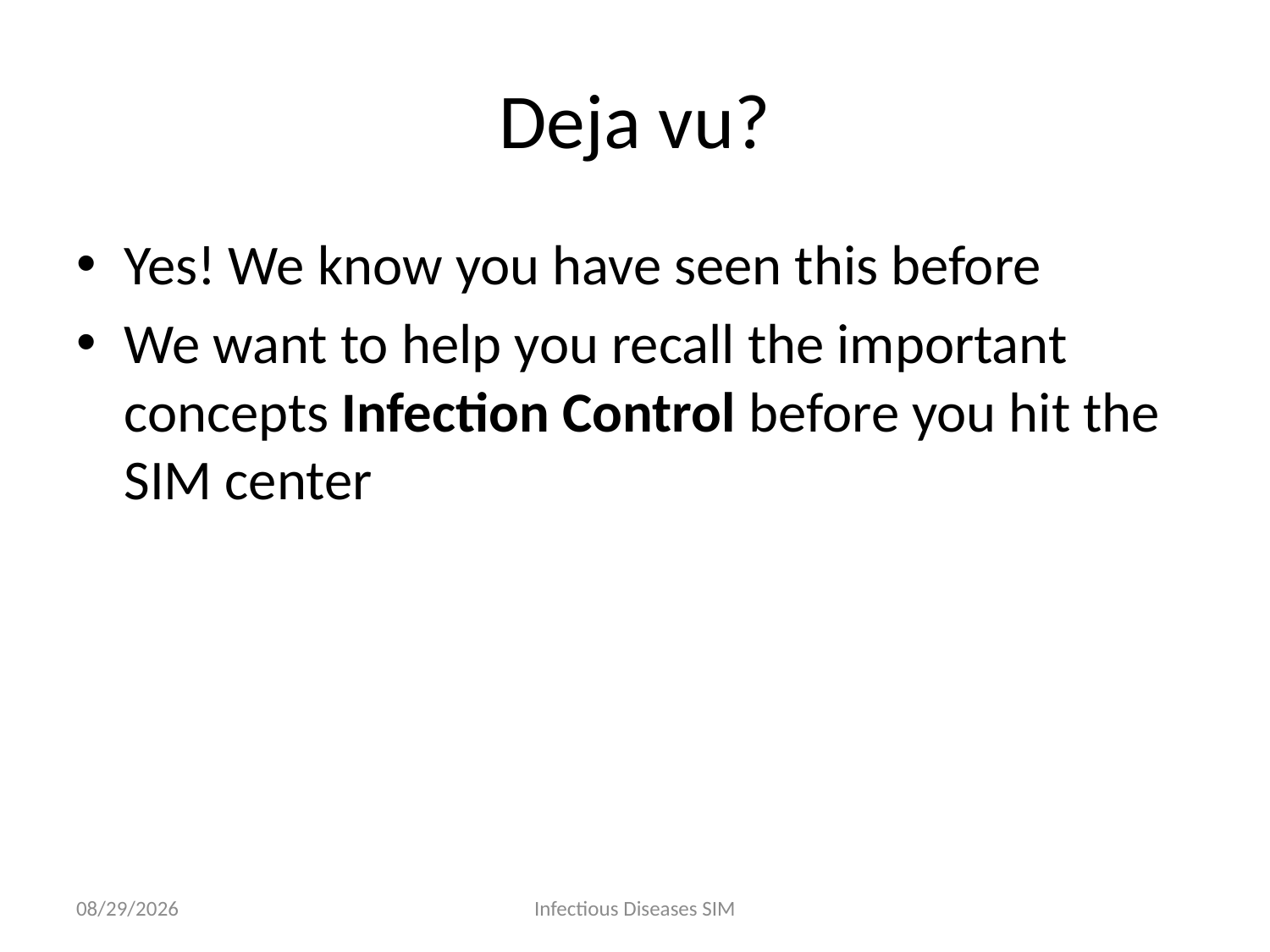

# Deja vu?
Yes! We know you have seen this before
We want to help you recall the important concepts Infection Control before you hit the SIM center
4/7/2020
Infectious Diseases SIM

## Slide 3
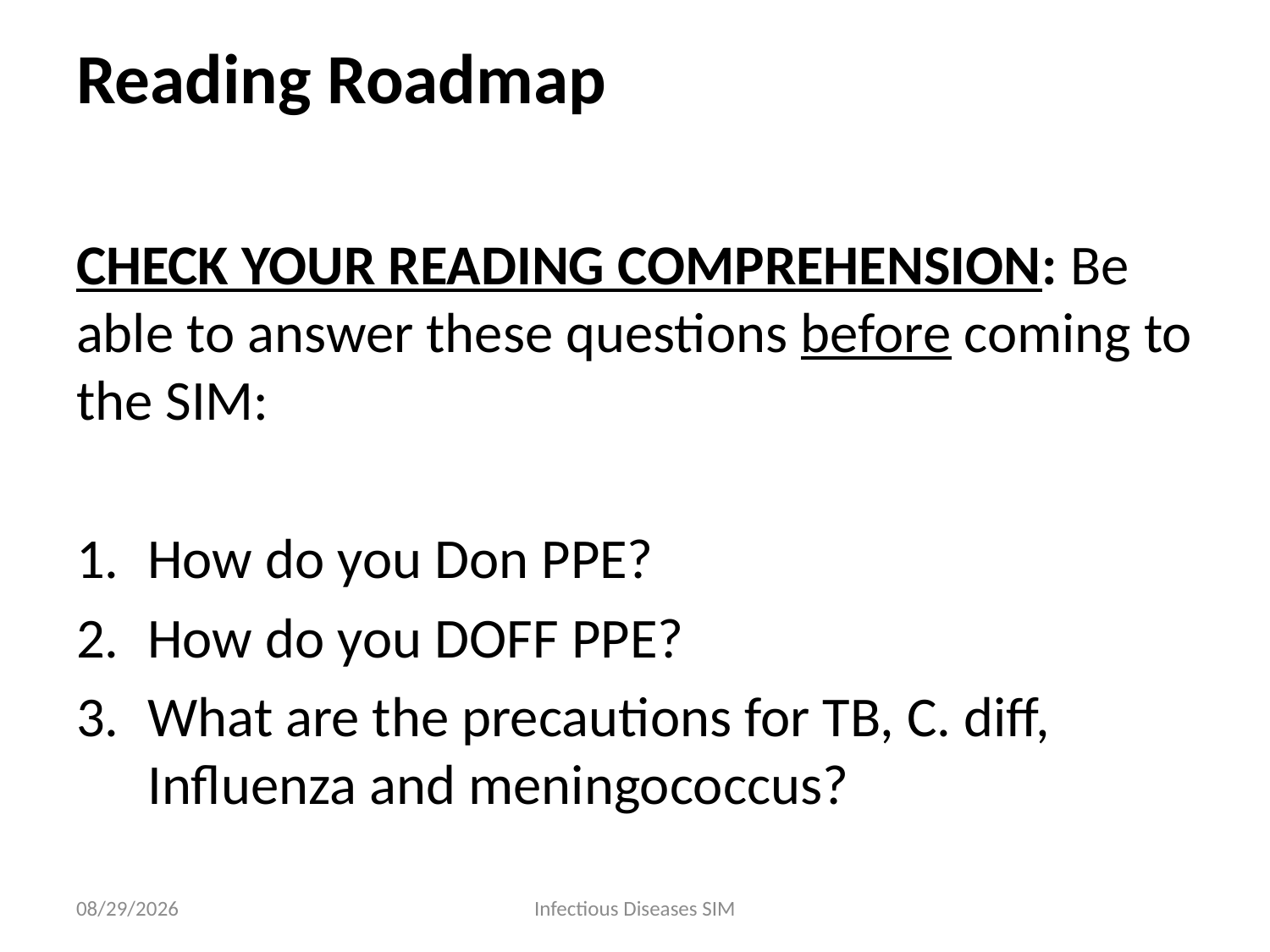

# Reading Roadmap
CHECK YOUR READING COMPREHENSION: Be able to answer these questions before coming to the SIM:
How do you Don PPE?
How do you DOFF PPE?
What are the precautions for TB, C. diff, Influenza and meningococcus?
4/7/2020
Infectious Diseases SIM

## Slide 4
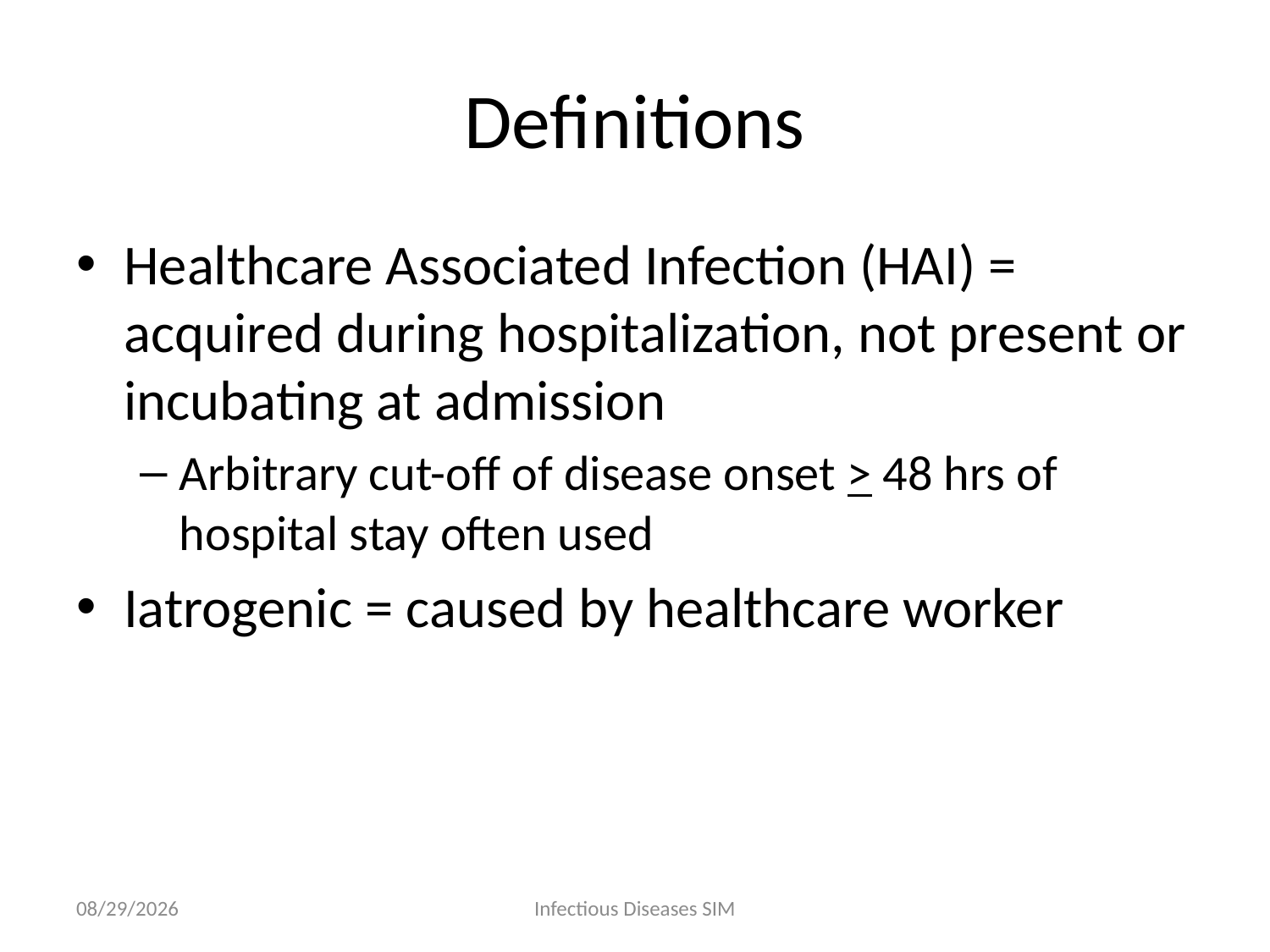

# Definitions
Healthcare Associated Infection (HAI) = acquired during hospitalization, not present or incubating at admission
Arbitrary cut-off of disease onset > 48 hrs of hospital stay often used
Iatrogenic = caused by healthcare worker
4/7/2020
Infectious Diseases SIM

## Slide 5
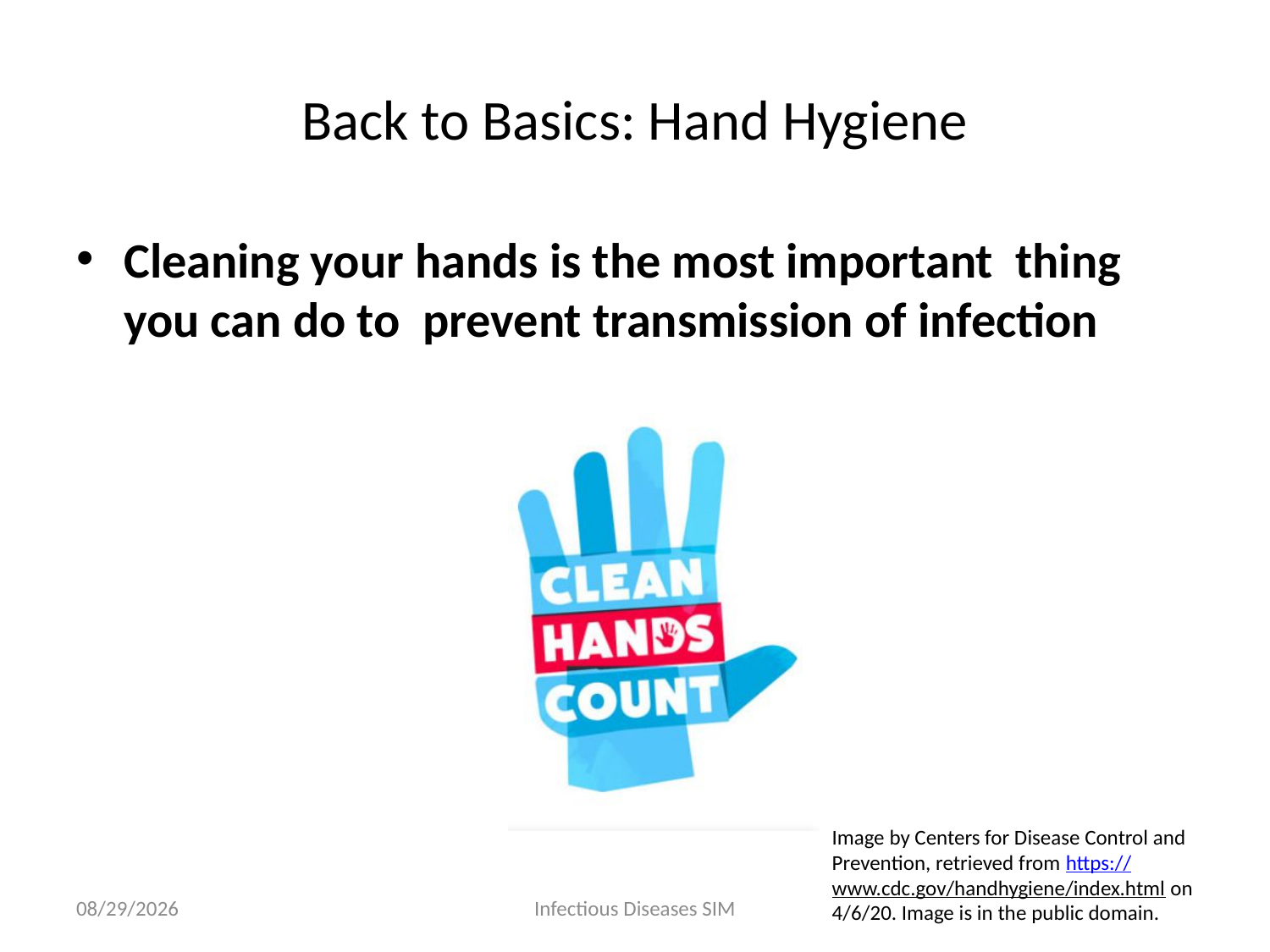

# Back to Basics: Hand Hygiene
Cleaning your hands is the most important thing you can do to prevent transmission of infection
Image by Centers for Disease Control and Prevention, retrieved from https://www.cdc.gov/handhygiene/index.html on 4/6/20. Image is in the public domain.
4/7/2020
Infectious Diseases SIM

## Slide 6
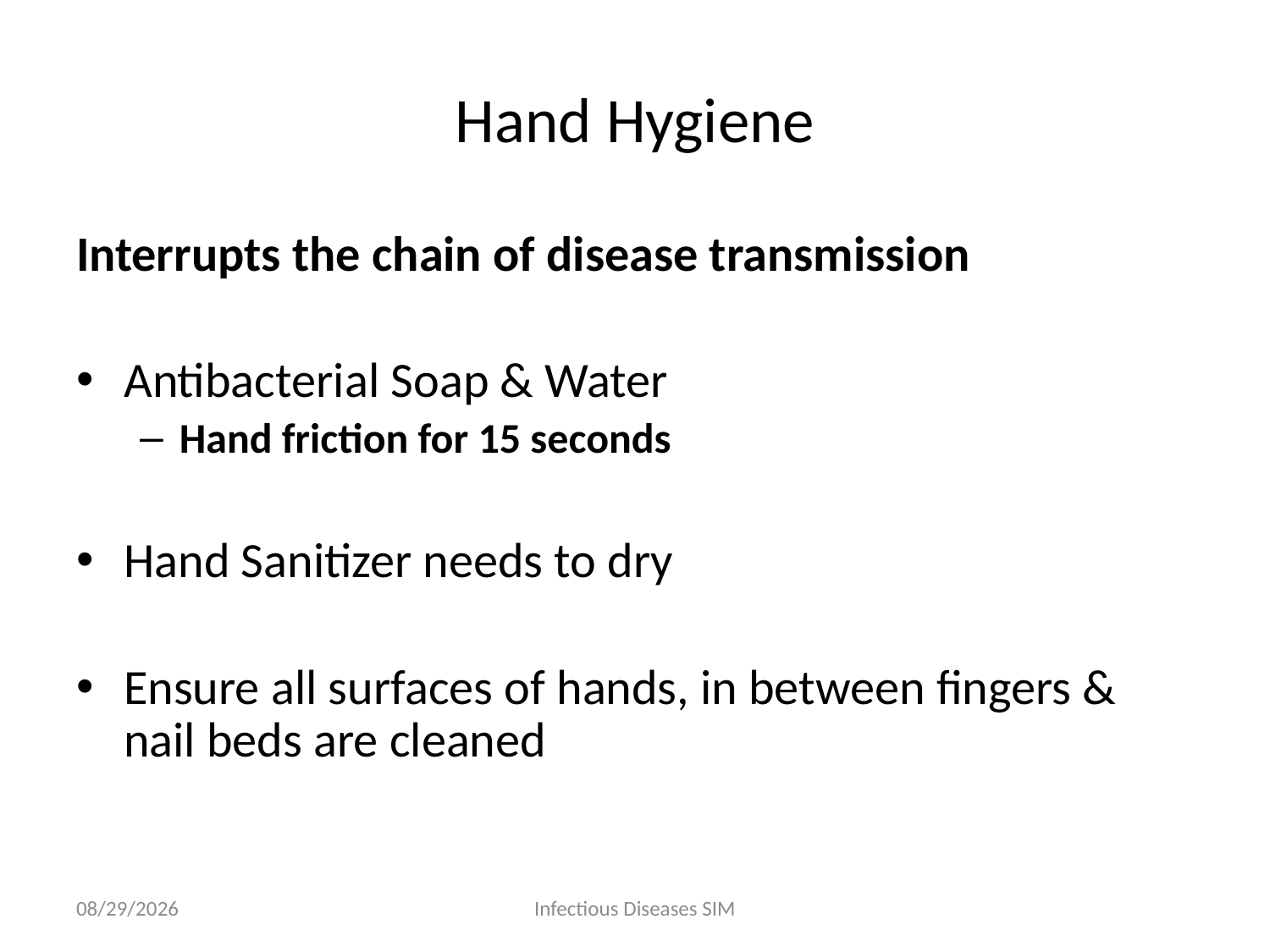

# Hand Hygiene
Interrupts the chain of disease transmission
Antibacterial Soap & Water
Hand friction for 15 seconds
Hand Sanitizer needs to dry
Ensure all surfaces of hands, in between fingers & nail beds are cleaned
4/7/2020
Infectious Diseases SIM

## Slide 7
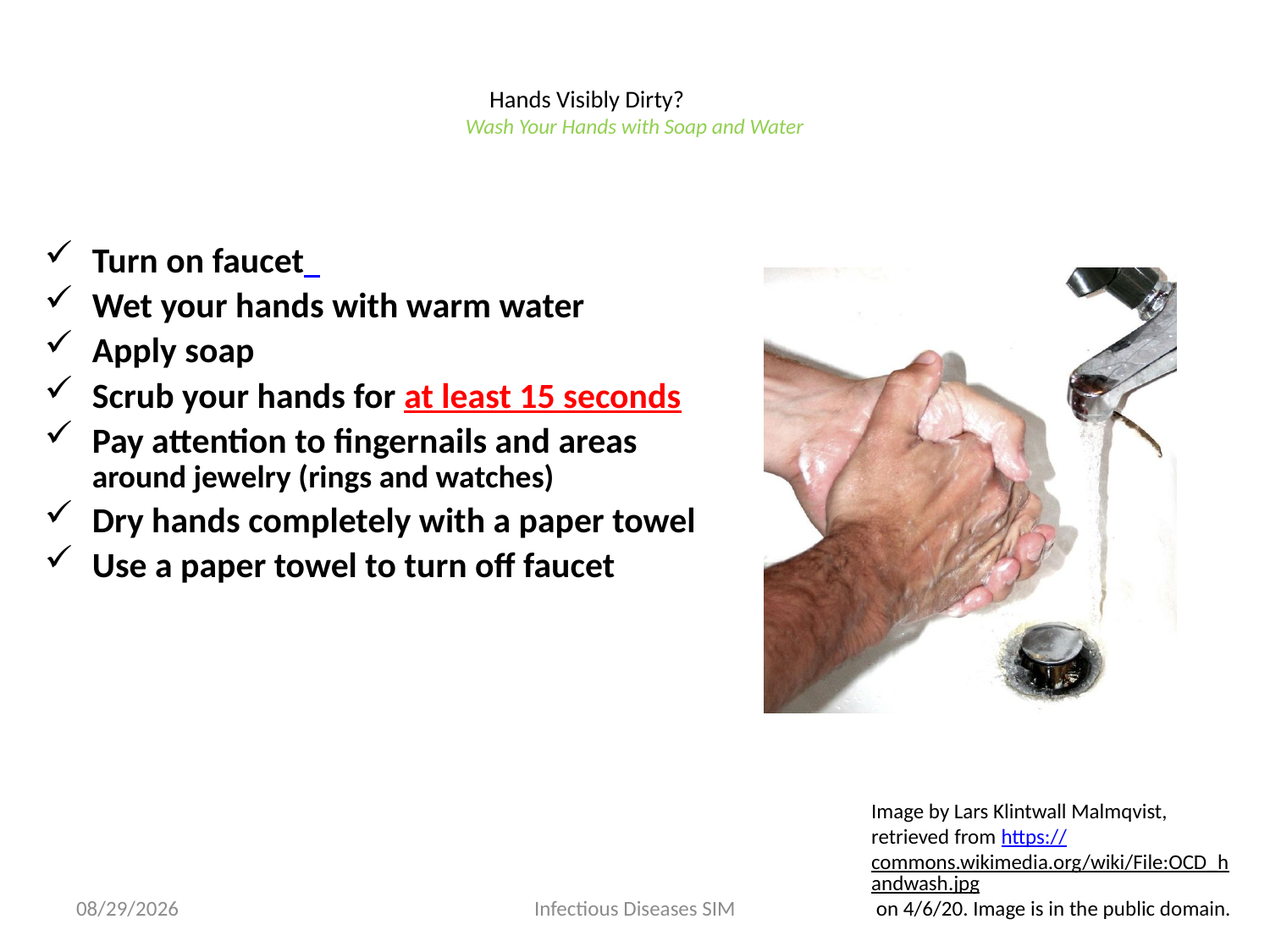

# Hands Visibly Dirty? Wash Your Hands with Soap and Water
Turn on faucet
Wet your hands with warm water
Apply soap
Scrub your hands for at least 15 seconds
Pay attention to fingernails and areas around jewelry (rings and watches)
Dry hands completely with a paper towel
Use a paper towel to turn off faucet
Image by Lars Klintwall Malmqvist, retrieved from https://commons.wikimedia.org/wiki/File:OCD_handwash.jpg on 4/6/20. Image is in the public domain.
4/7/2020
Infectious Diseases SIM

## Slide 8
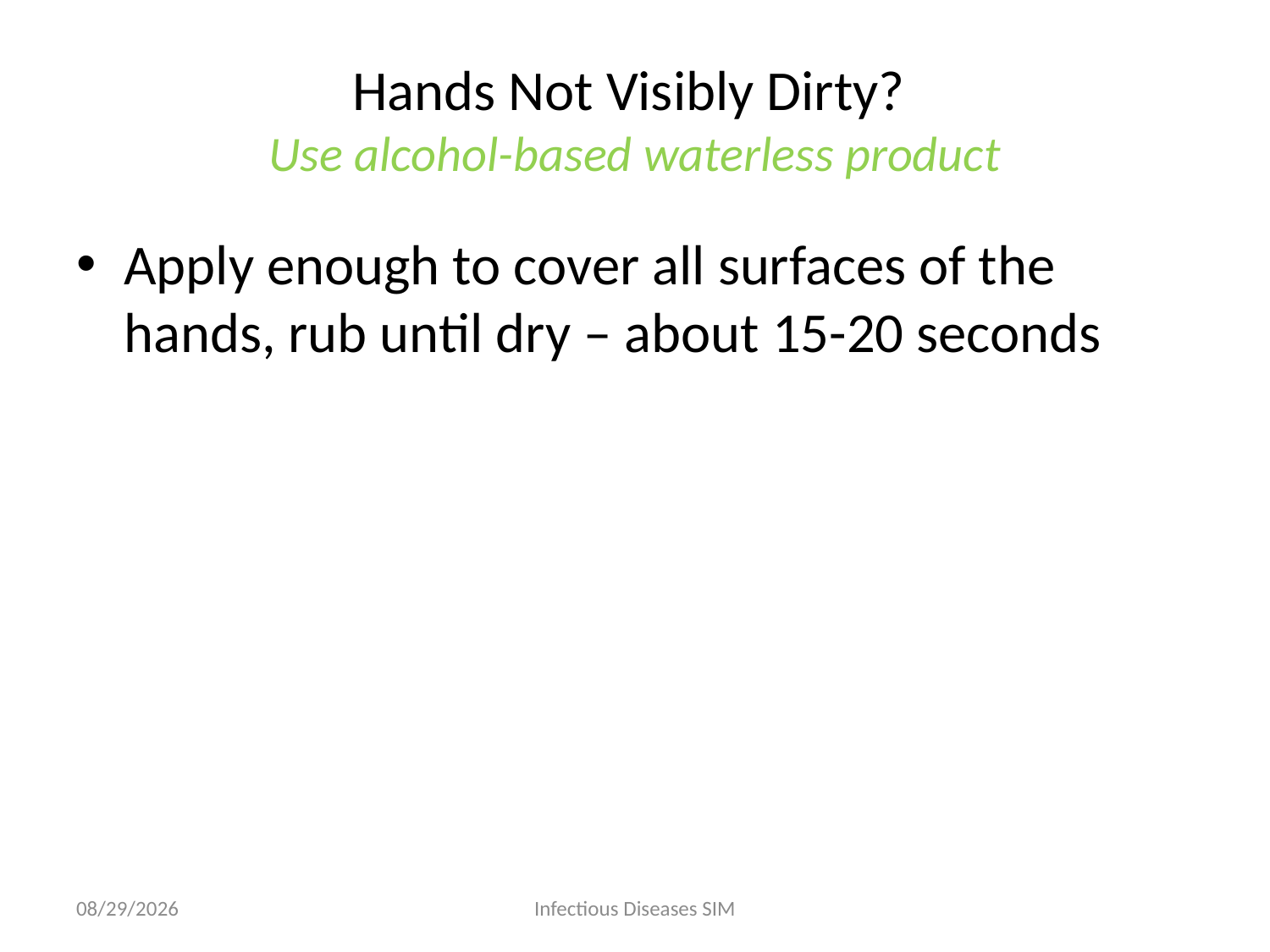

# Hands Not Visibly Dirty? Use alcohol-based waterless product
Apply enough to cover all surfaces of the hands, rub until dry – about 15-20 seconds
4/7/2020
Infectious Diseases SIM

## Slide 9
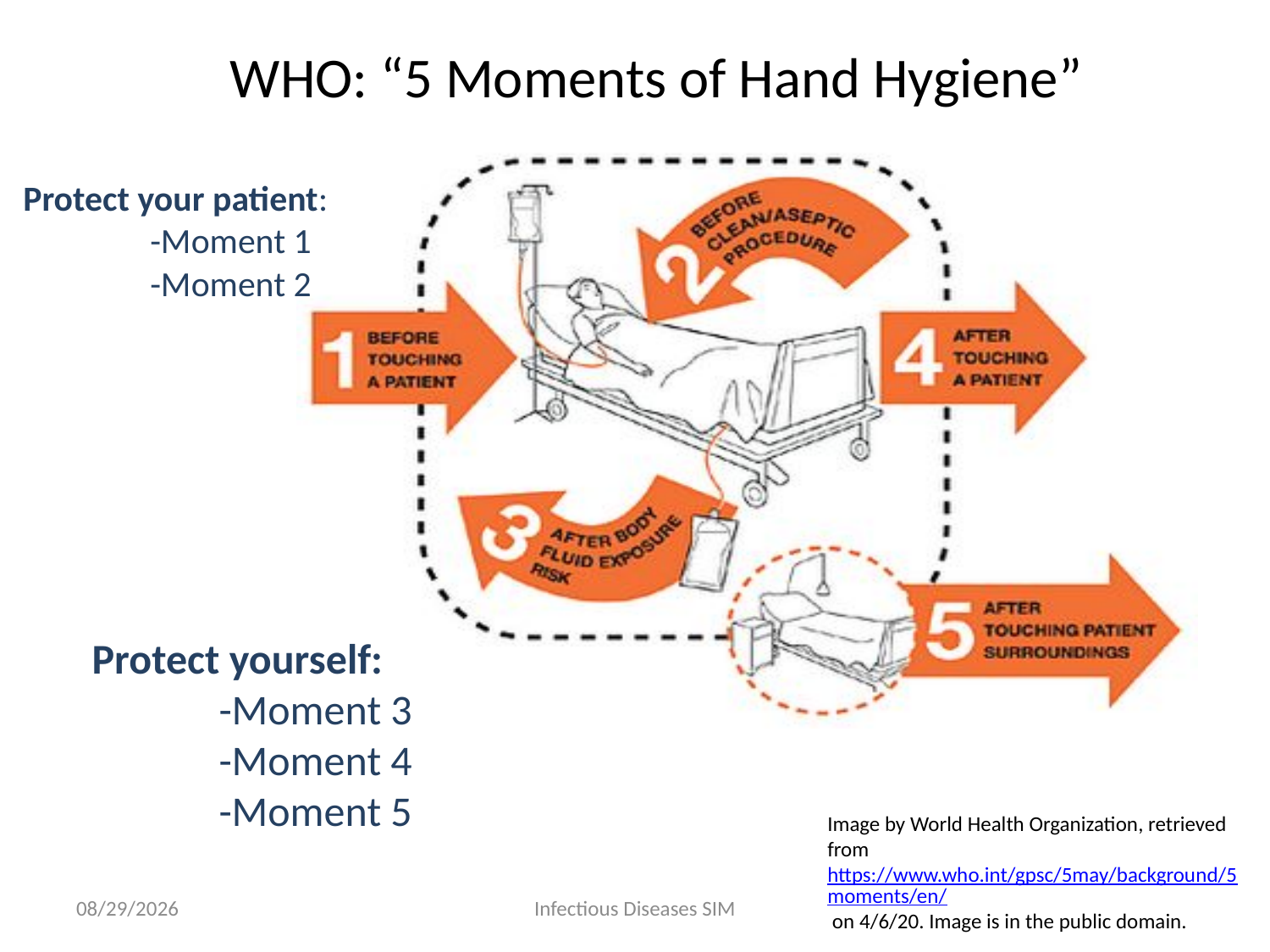

# WHO: “5 Moments of Hand Hygiene”
Protect your patient:
	-Moment 1
	-Moment 2
Protect yourself:
	-Moment 3
	-Moment 4
	-Moment 5
Image by World Health Organization, retrieved from https://www.who.int/gpsc/5may/background/5moments/en/ on 4/6/20. Image is in the public domain.
4/7/2020
Infectious Diseases SIM

## Slide 10
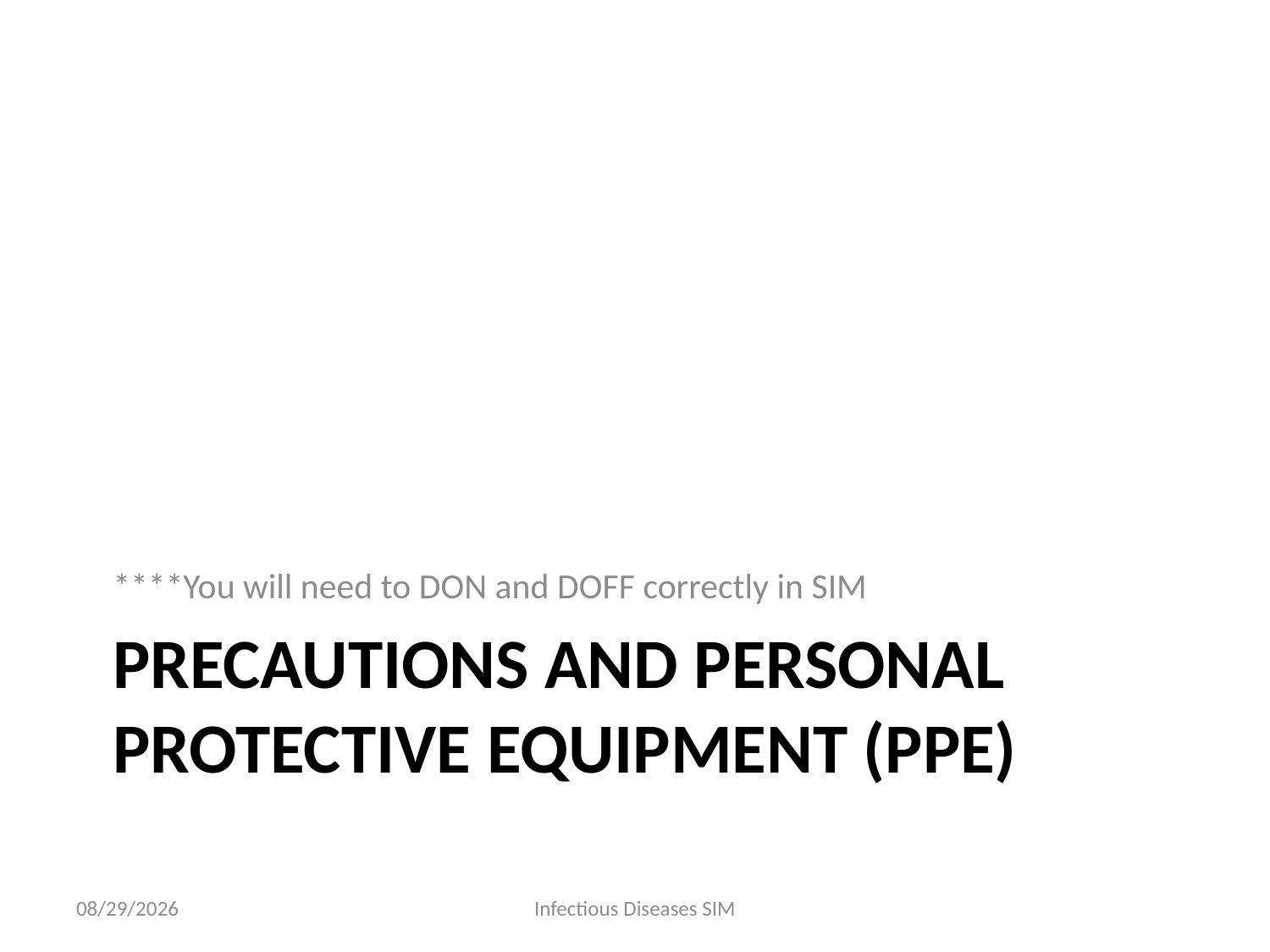

****You will need to DON and DOFF correctly in SIM
# Precautions and Personal Protective Equipment (PPE)
4/7/2020
Infectious Diseases SIM

## Slide 11
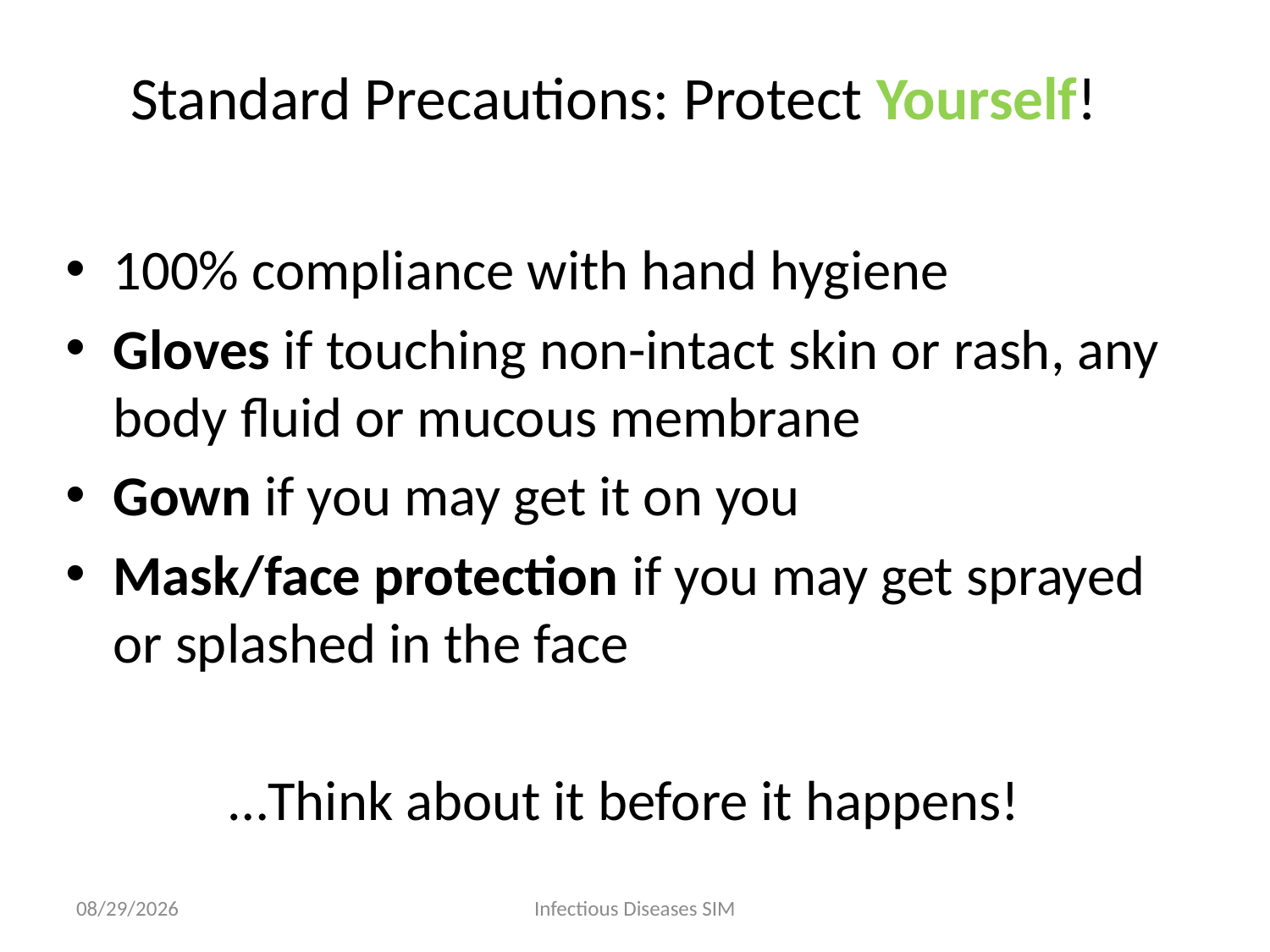

# Standard Precautions: Protect Yourself!
100% compliance with hand hygiene
Gloves if touching non-intact skin or rash, any body fluid or mucous membrane
Gown if you may get it on you
Mask/face protection if you may get sprayed or splashed in the face
…Think about it before it happens!
4/7/2020
Infectious Diseases SIM

## Slide 12
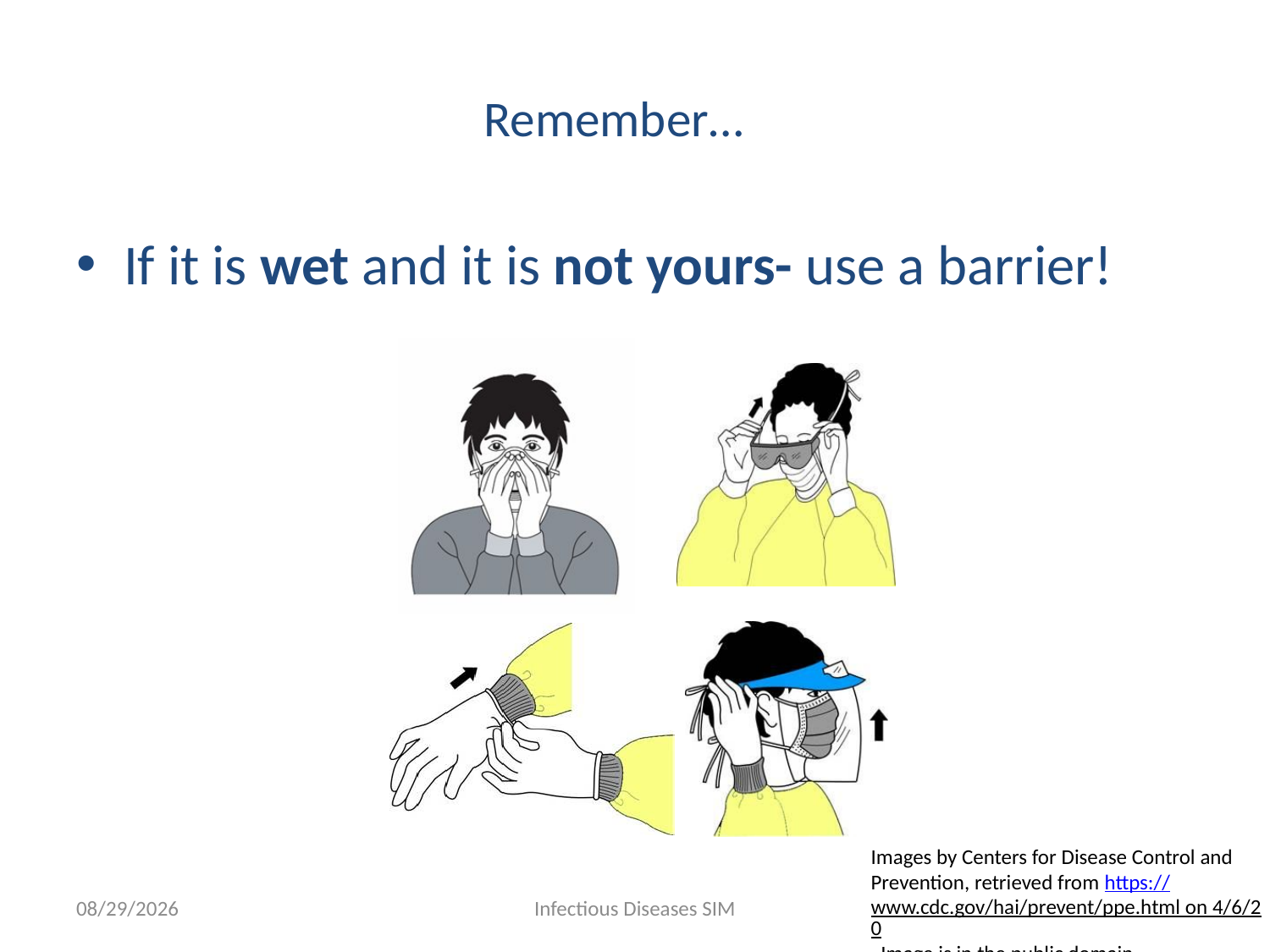

# Remember…
If it is wet and it is not yours- use a barrier!
Images by Centers for Disease Control and Prevention, retrieved from https://www.cdc.gov/hai/prevent/ppe.html on 4/6/20. Image is in the public domain.
4/7/2020
Infectious Diseases SIM

## Slide 13
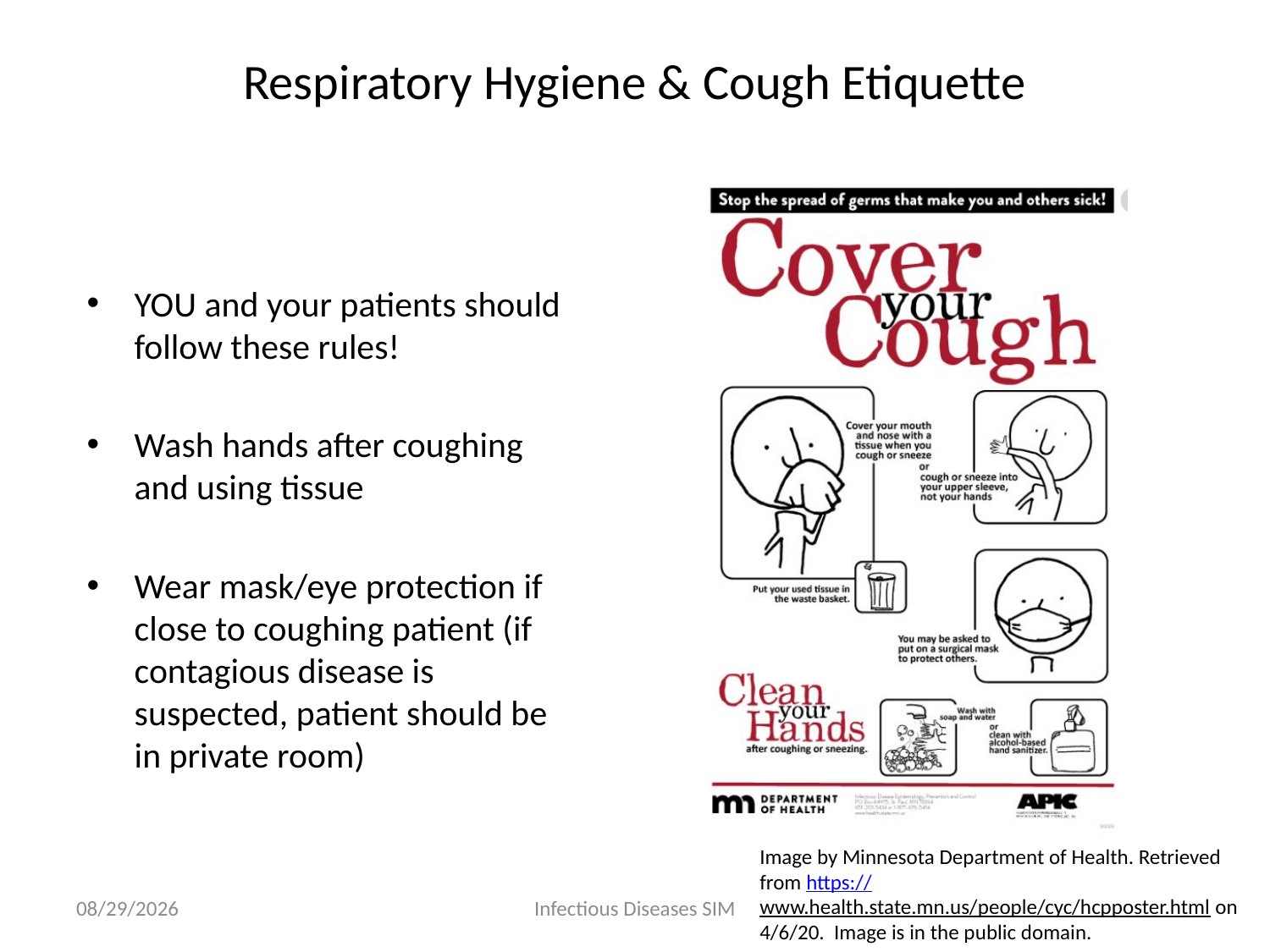

# Respiratory Hygiene & Cough Etiquette
YOU and your patients should follow these rules!
Wash hands after coughing and using tissue
Wear mask/eye protection if close to coughing patient (if contagious disease is suspected, patient should be in private room)
Image by Minnesota Department of Health. Retrieved from https://www.health.state.mn.us/people/cyc/hcpposter.html on 4/6/20. Image is in the public domain.
4/7/2020
Infectious Diseases SIM

## Slide 14
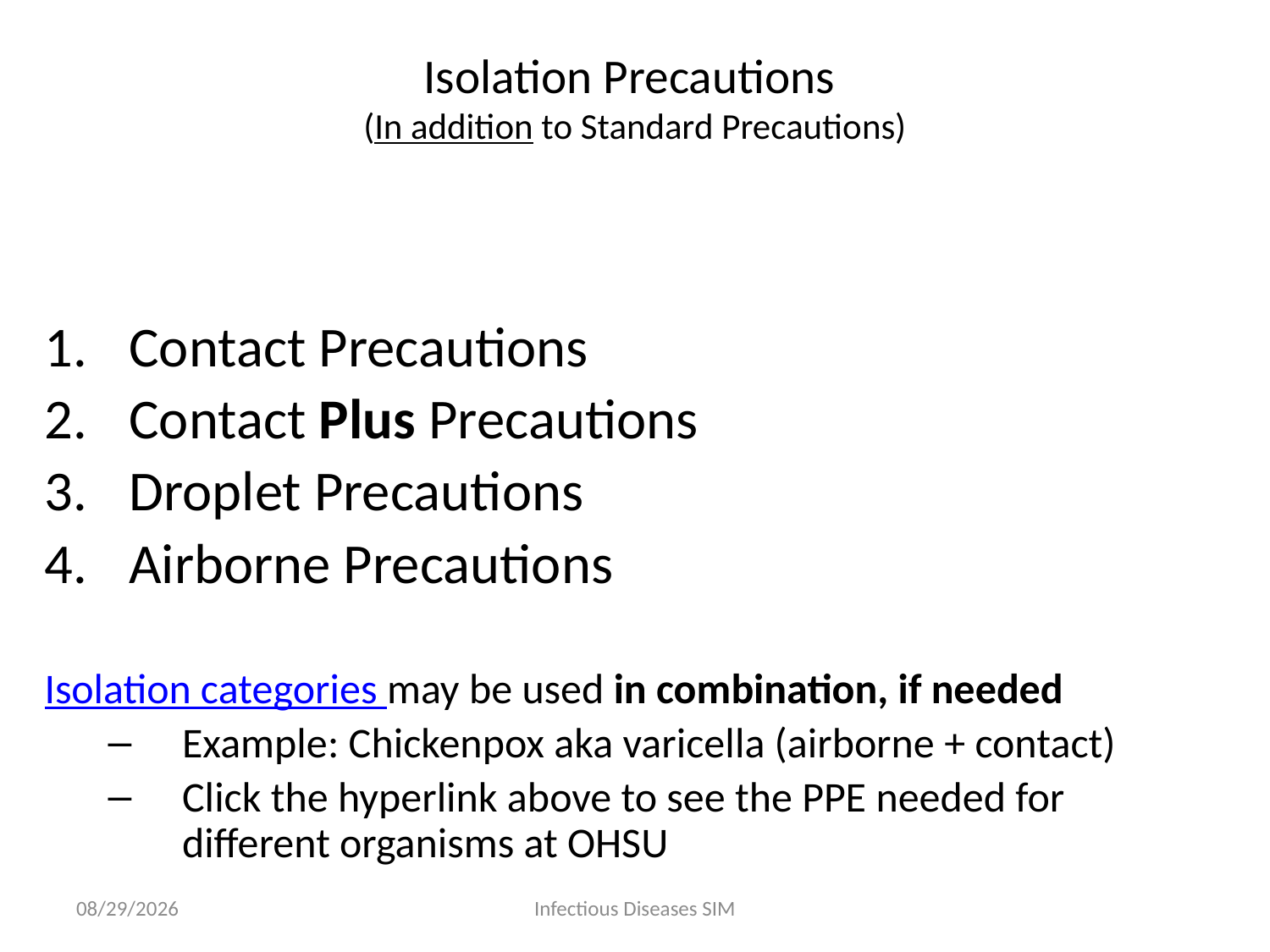

# Isolation Precautions (In addition to Standard Precautions)
Contact Precautions
Contact Plus Precautions
Droplet Precautions
Airborne Precautions
Isolation categories may be used in combination, if needed
Example: Chickenpox aka varicella (airborne + contact)
Click the hyperlink above to see the PPE needed for different organisms at OHSU
4/7/2020
Infectious Diseases SIM

## Slide 15
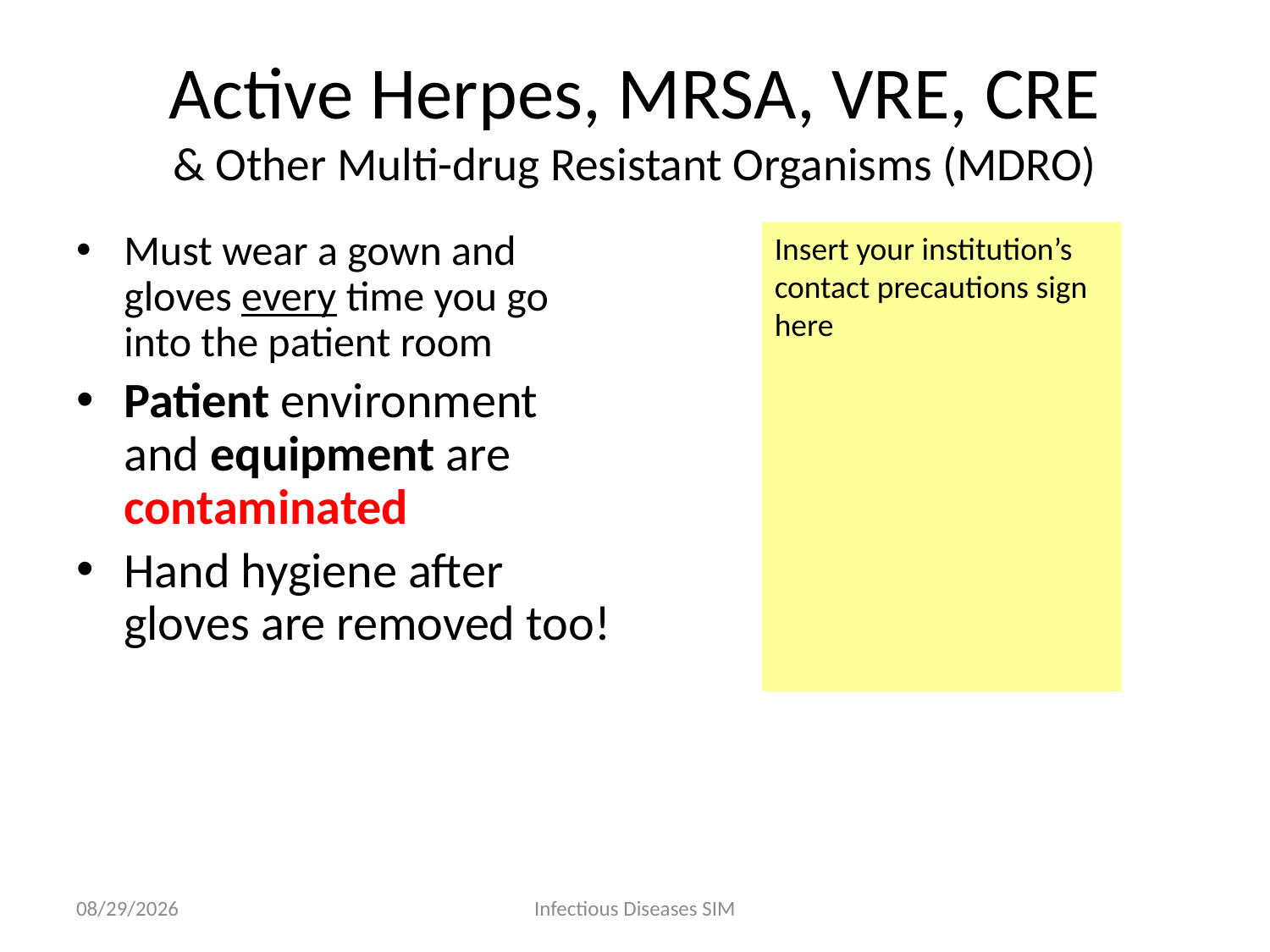

# Active Herpes, MRSA, VRE, CRE & Other Multi-drug Resistant Organisms (MDRO)
Must wear a gown and gloves every time you go into the patient room
Patient environment and equipment are contaminated
Hand hygiene after gloves are removed too!
Insert your institution’s contact precautions sign here
4/7/2020
Infectious Diseases SIM

## Slide 16
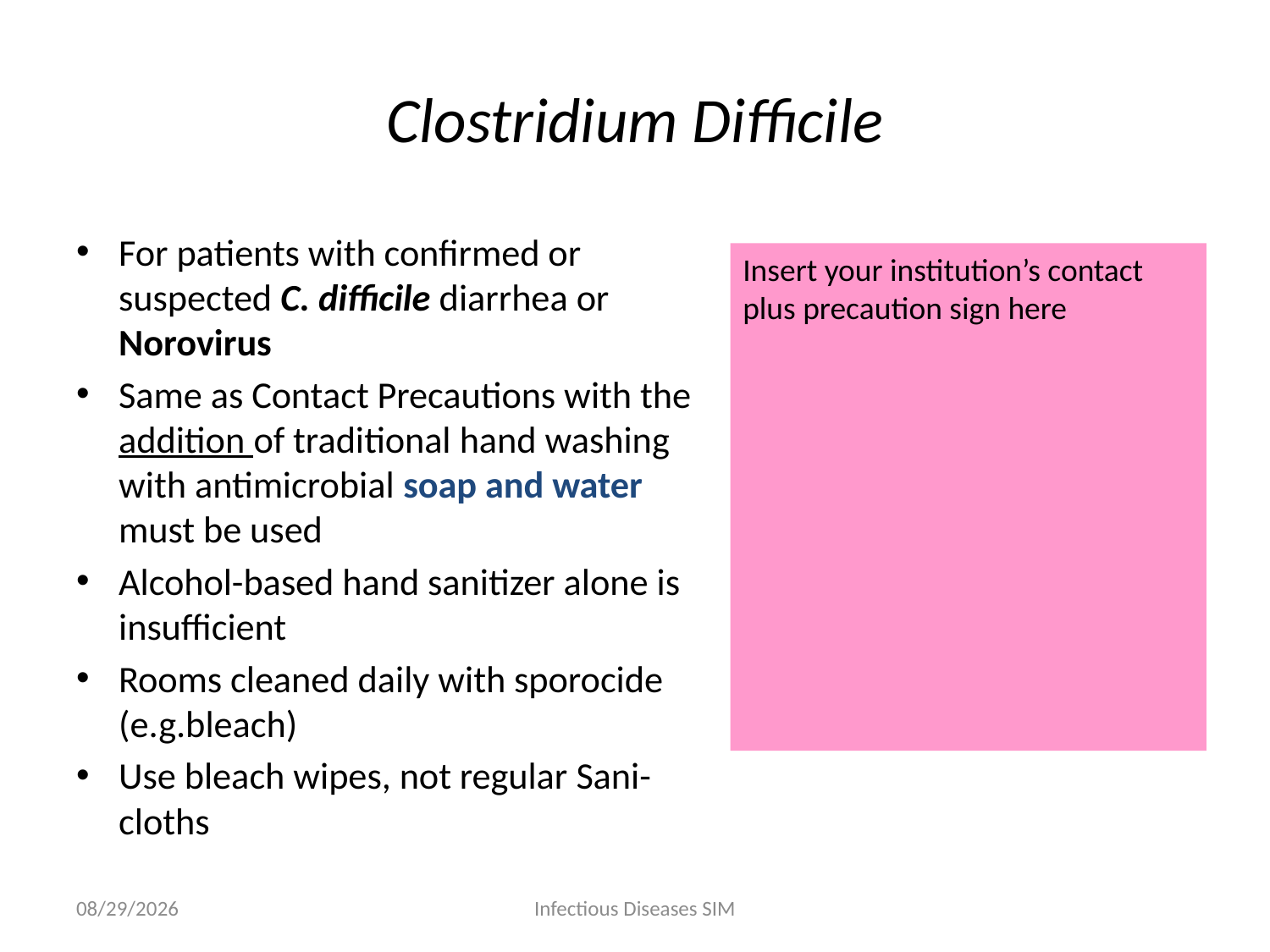

# Clostridium Difficile
For patients with confirmed or suspected C. difficile diarrhea or Norovirus
Same as Contact Precautions with the addition of traditional hand washing with antimicrobial soap and water must be used
Alcohol-based hand sanitizer alone is insufficient
Rooms cleaned daily with sporocide (e.g.bleach)
Use bleach wipes, not regular Sani-cloths
Insert your institution’s contact plus precaution sign here
4/7/2020
Infectious Diseases SIM

## Slide 17
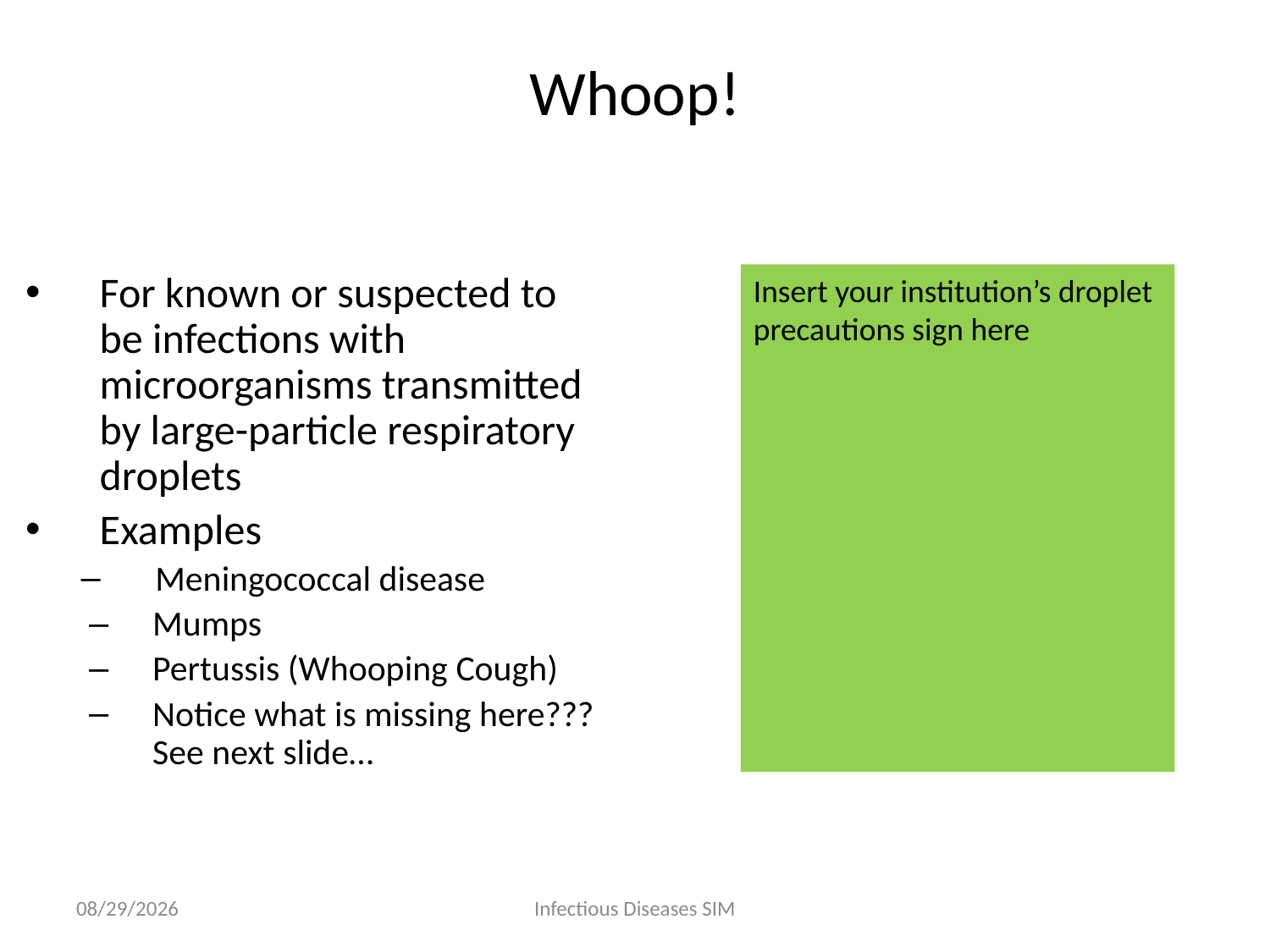

# Whoop!
For known or suspected to be infections with microorganisms transmitted by large-particle respiratory droplets
Examples
Meningococcal disease
Mumps
Pertussis (Whooping Cough)
Notice what is missing here??? See next slide…
Insert your institution’s droplet precautions sign here
4/7/2020
Infectious Diseases SIM

## Slide 18
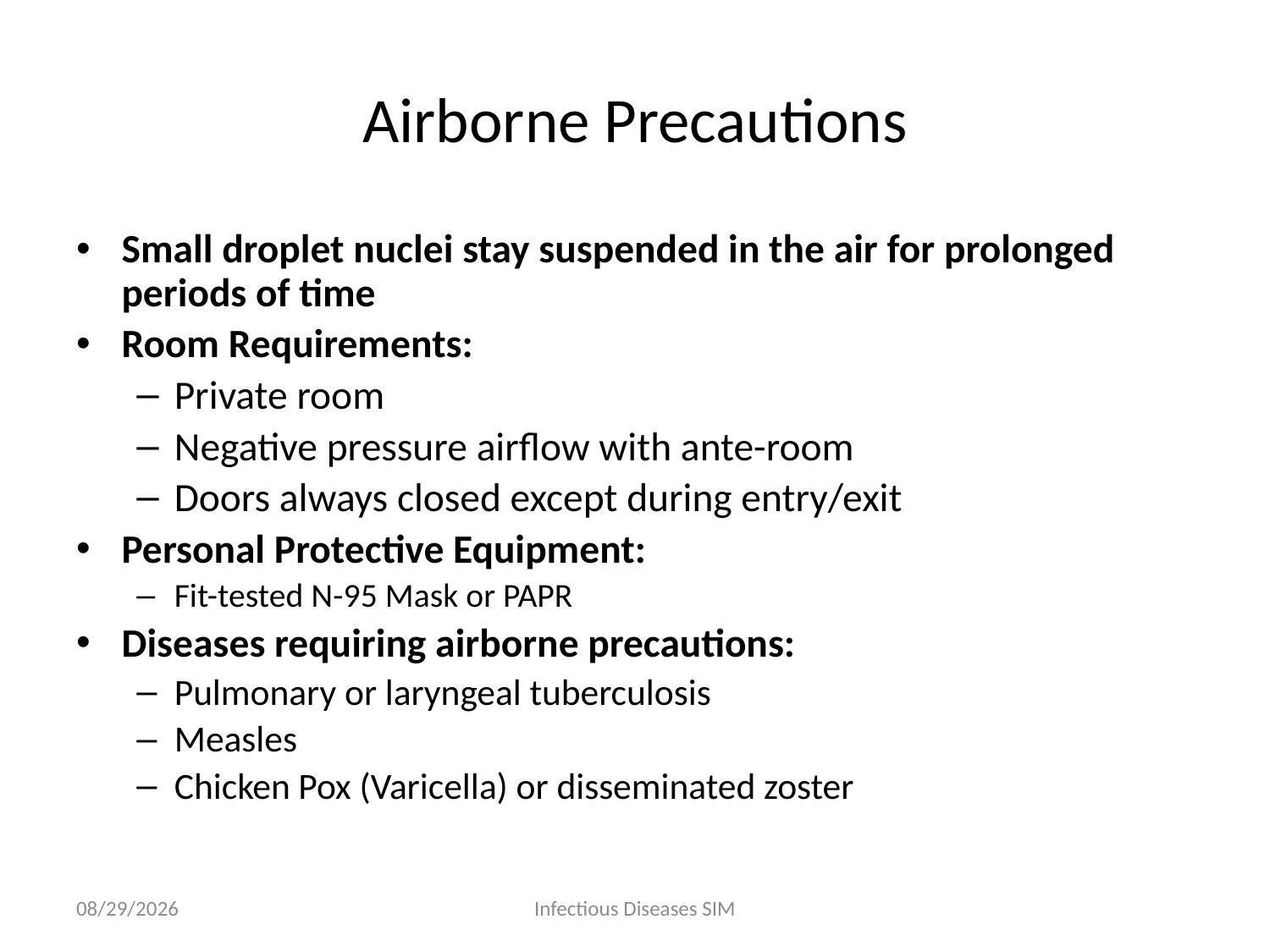

# Airborne Precautions
Small droplet nuclei stay suspended in the air for prolonged periods of time
Room Requirements:
Private room
Negative pressure airflow with ante-room
Doors always closed except during entry/exit
Personal Protective Equipment:
Fit-tested N-95 Mask or PAPR
Diseases requiring airborne precautions:
Pulmonary or laryngeal tuberculosis
Measles
Chicken Pox (Varicella) or disseminated zoster
4/7/2020
Infectious Diseases SIM

## Slide 19
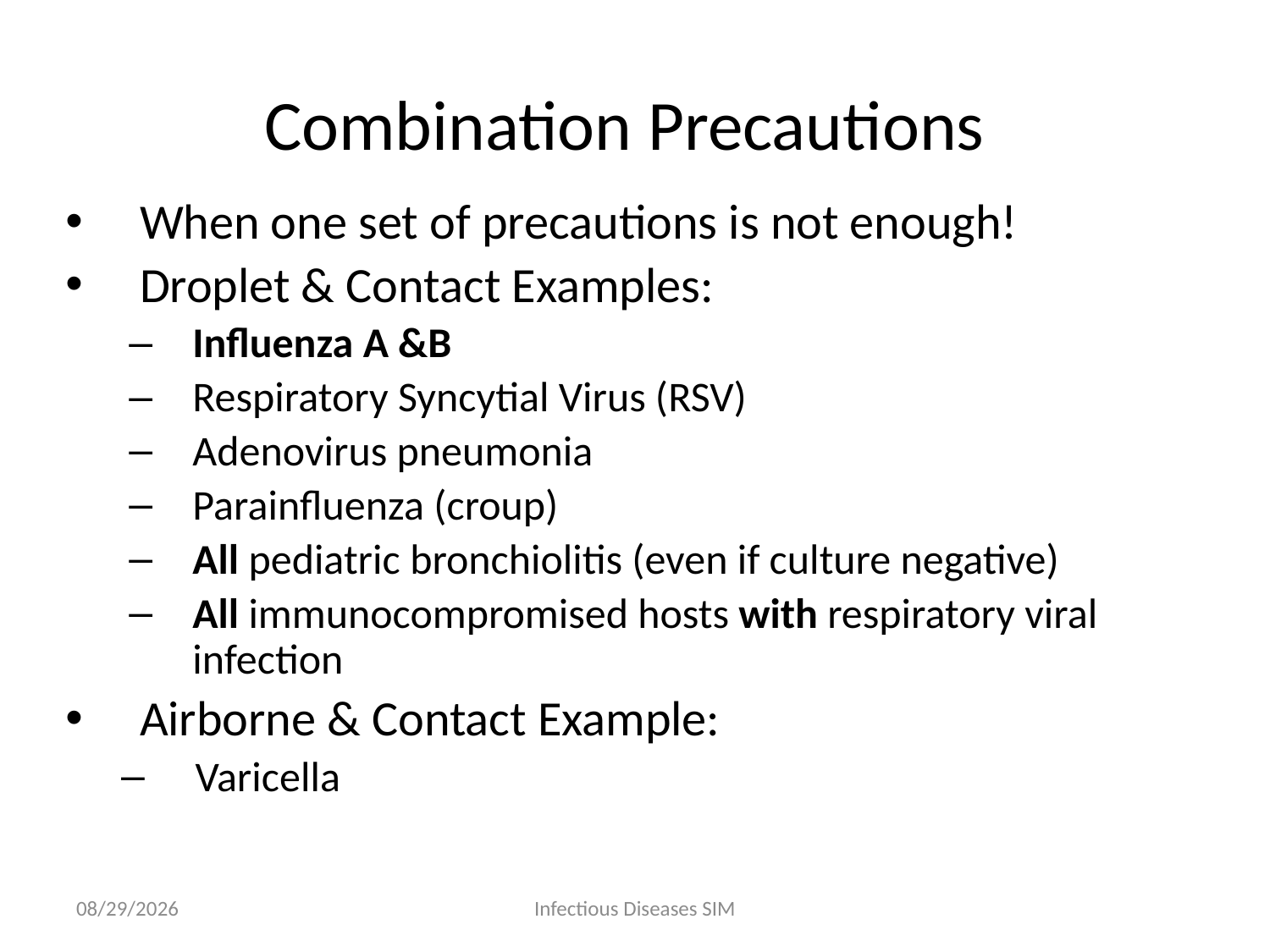

# Combination Precautions
When one set of precautions is not enough!
Droplet & Contact Examples:
Influenza A &B
Respiratory Syncytial Virus (RSV)
Adenovirus pneumonia
Parainfluenza (croup)
All pediatric bronchiolitis (even if culture negative)
All immunocompromised hosts with respiratory viral infection
Airborne & Contact Example:
Varicella
4/7/2020
Infectious Diseases SIM

## Slide 20
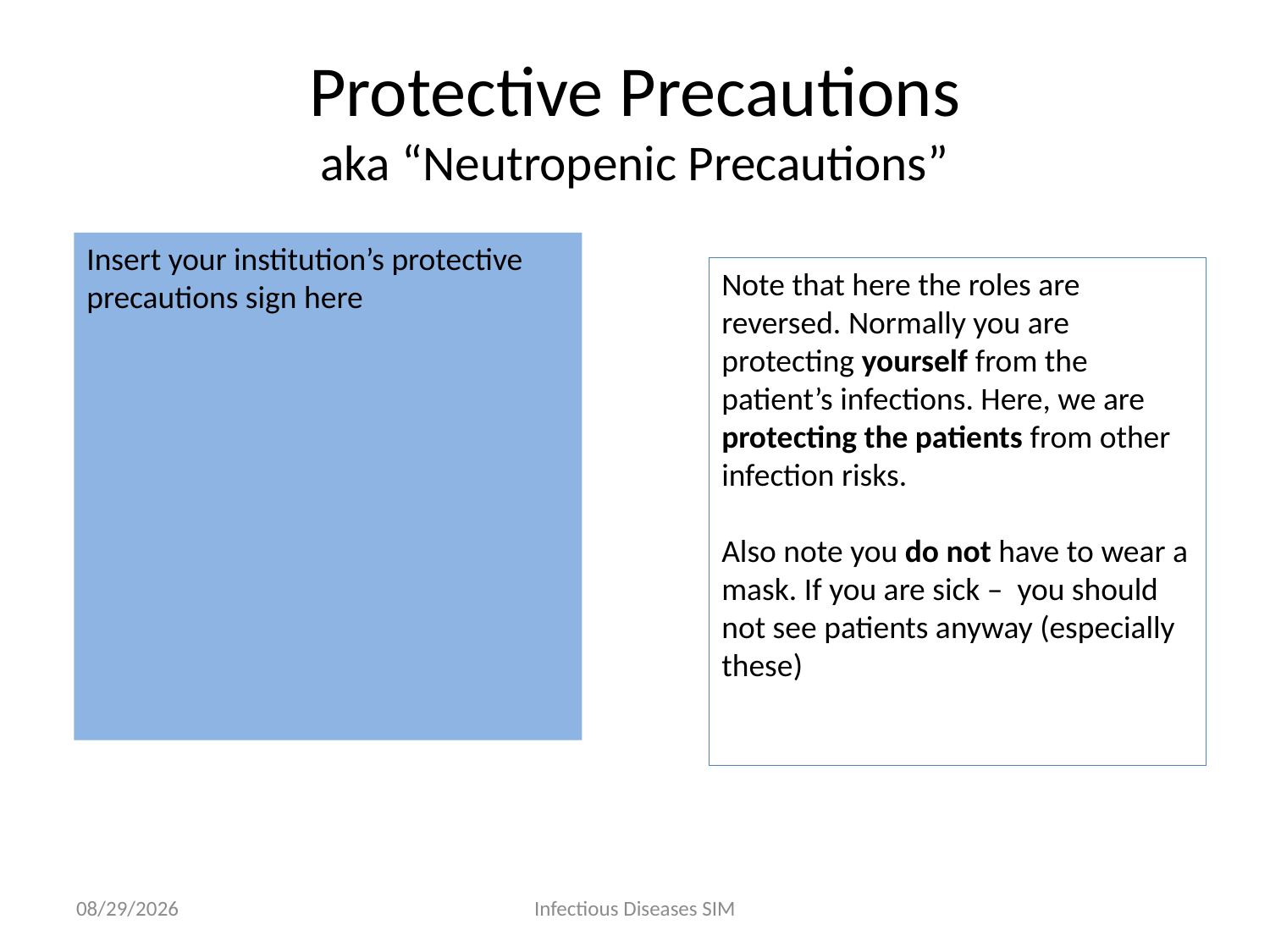

# Protective Precautions aka “Neutropenic Precautions”
Insert your institution’s protective precautions sign here
Note that here the roles are reversed. Normally you are protecting yourself from the patient’s infections. Here, we are protecting the patients from other infection risks.
Also note you do not have to wear a mask. If you are sick – you should not see patients anyway (especially these)
4/7/2020
Infectious Diseases SIM

## Slide 21
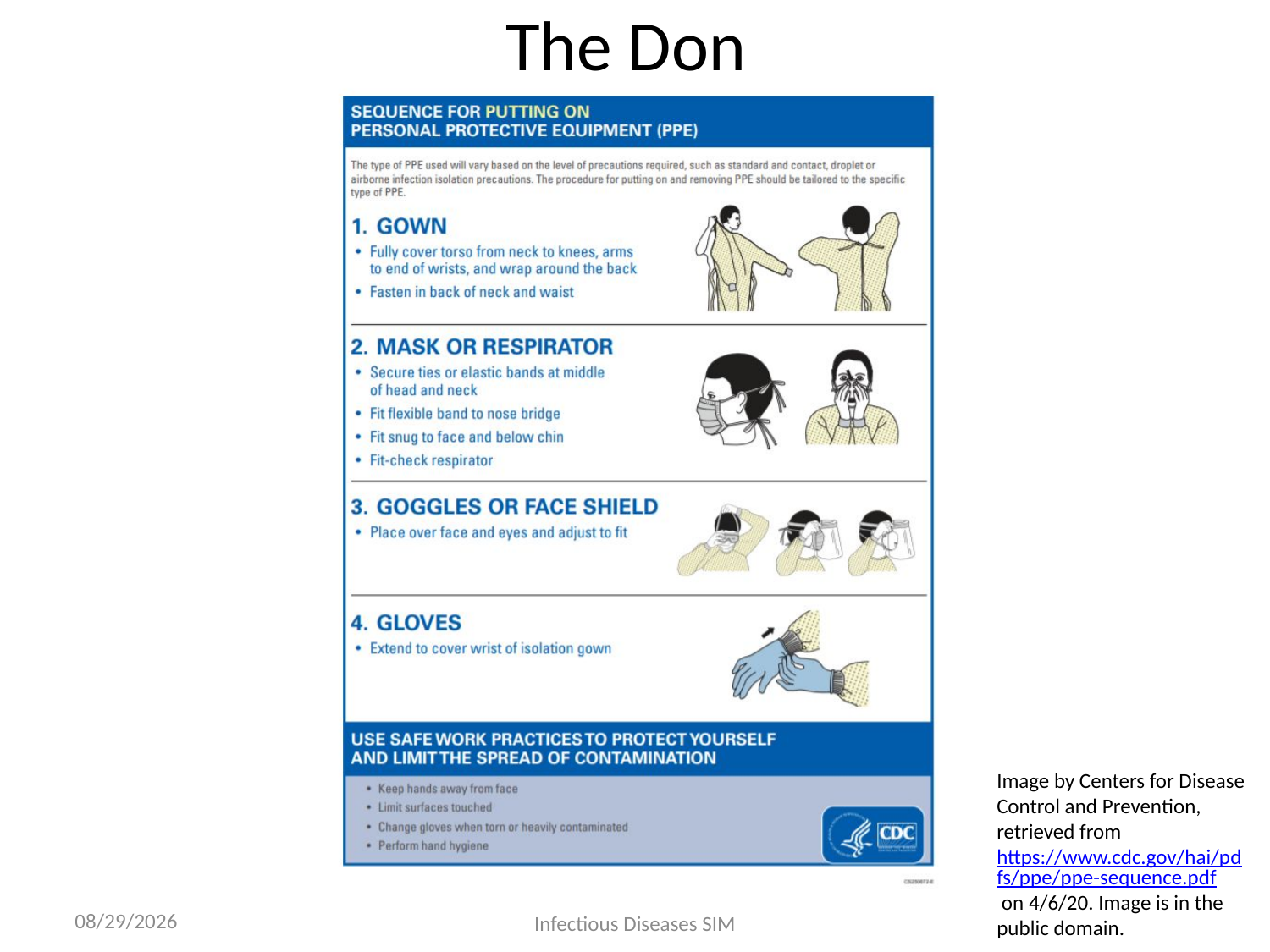

# The Don
Image by Centers for Disease Control and Prevention, retrieved from https://www.cdc.gov/hai/pdfs/ppe/ppe-sequence.pdf on 4/6/20. Image is in the public domain.
4/7/2020
Infectious Diseases SIM

## Slide 22
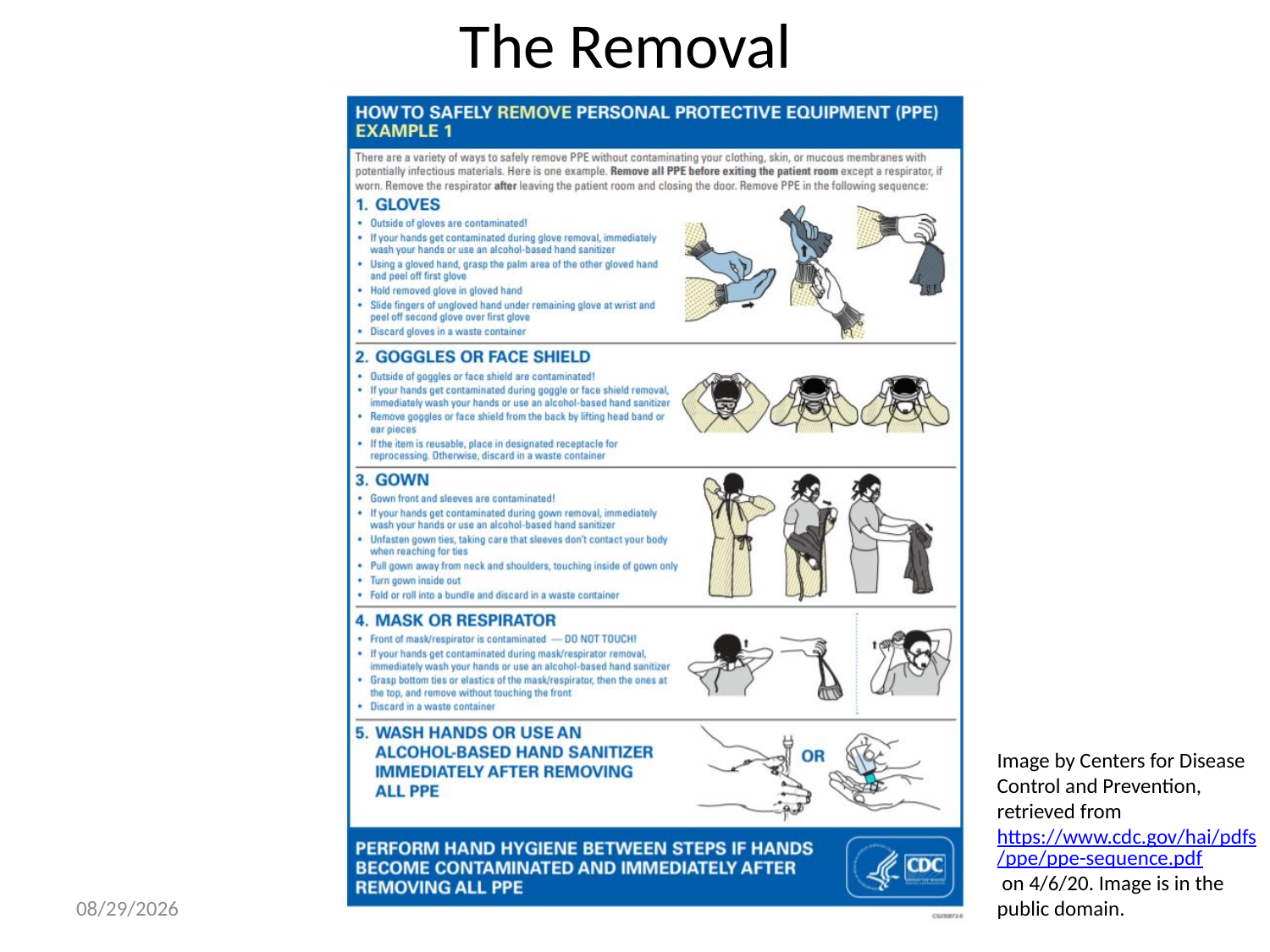

# The Removal
Image by Centers for Disease Control and Prevention, retrieved from https://www.cdc.gov/hai/pdfs/ppe/ppe-sequence.pdf on 4/6/20. Image is in the public domain.
4/7/2020
Infectious Diseases SIM

## Slide 23
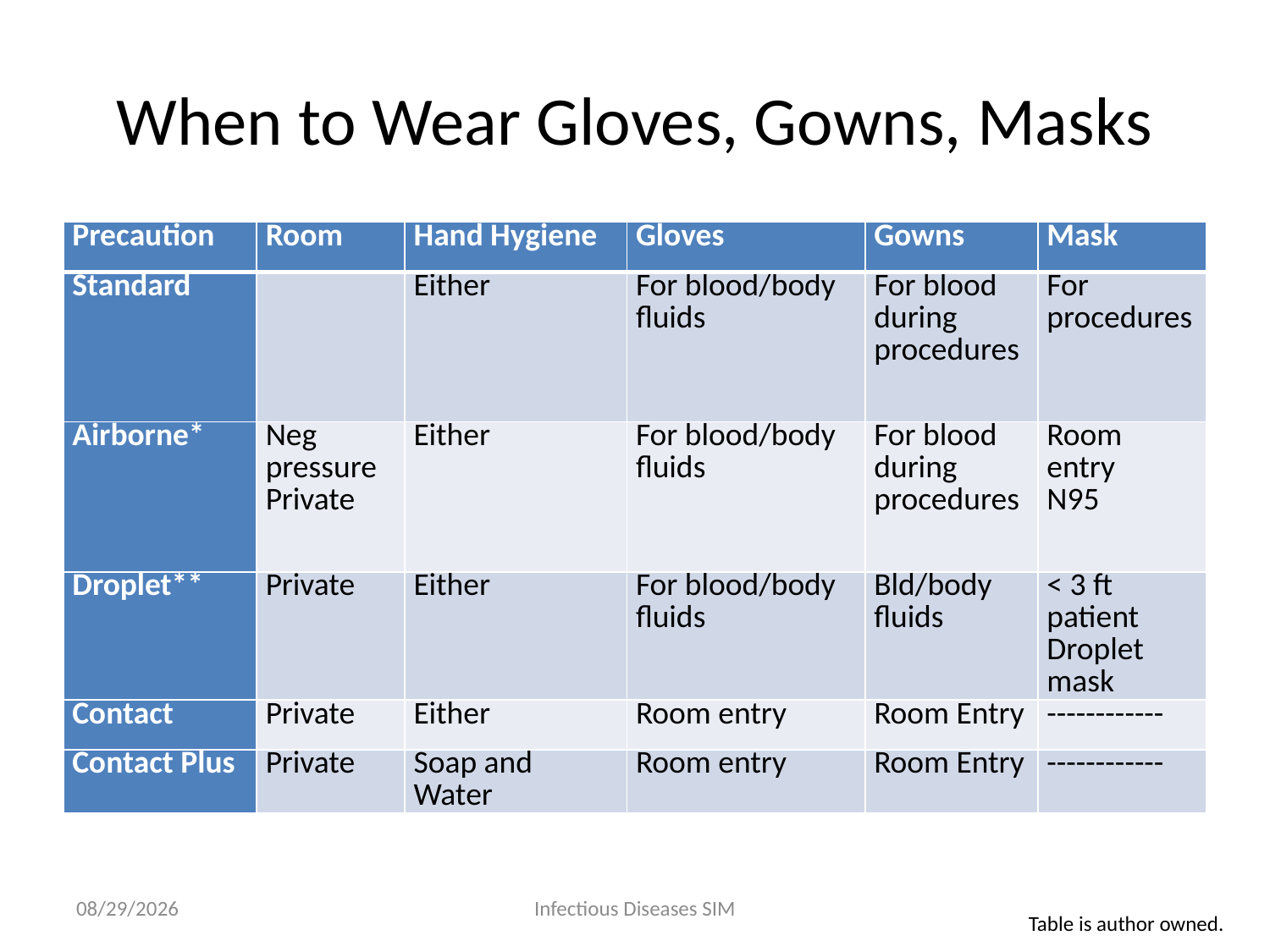

# When to Wear Gloves, Gowns, Masks
| Precaution | Room | Hand Hygiene | Gloves | Gowns | Mask |
| --- | --- | --- | --- | --- | --- |
| Standard | | Either | For blood/body fluids | For blood during procedures | For procedures |
| Airborne\* | Neg pressure Private | Either | For blood/body fluids | For blood during procedures | Room entry N95 |
| Droplet\*\* | Private | Either | For blood/body fluids | Bld/body fluids | < 3 ft patient Droplet mask |
| Contact | Private | Either | Room entry | Room Entry | ------------ |
| Contact Plus | Private | Soap and Water | Room entry | Room Entry | ------------ |
4/7/2020
Infectious Diseases SIM
Table is author owned.

## Slide 24
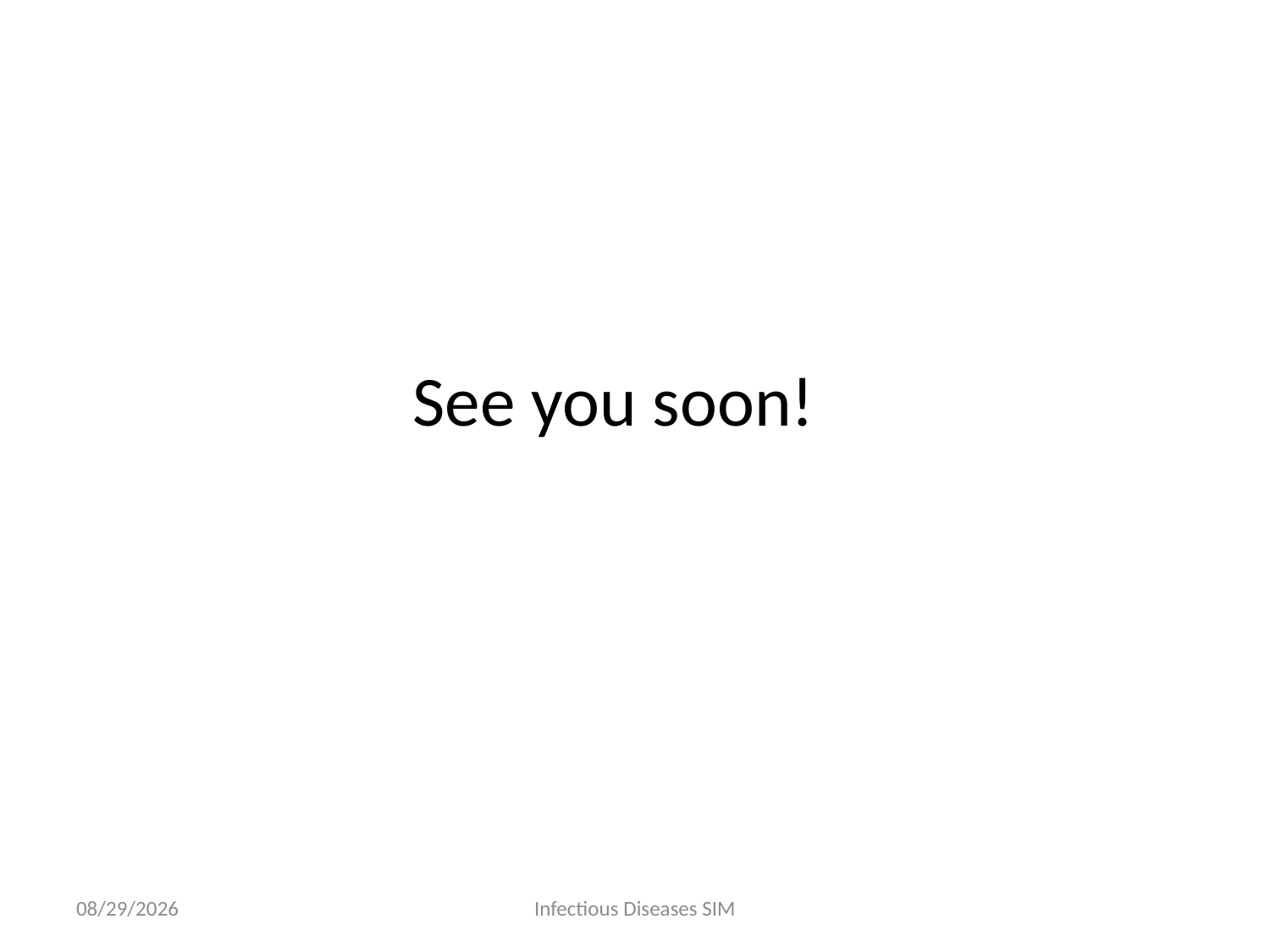

See you soon!
4/7/2020
Infectious Diseases SIM
