# Supplementary material for: Infectious Diseases Simulation for Medical Students: Experiential Instruction on Personal Protective Equipment
Source: MedEdPORTAL. 2020 Nov 24;16:11031. doi: 10.15766/mep_2374-8265.11031 (PMC7703477; doi:10.15766/mep_2374-8265.11031)
Supplement: Supplementary file 1 — Prework Slides.pptxSimulation Case 1.docxSimulation Case 2.docxSimulation Case 3.docxExam Questions.docxEvaluation Questions.docx [file mep_2374-8265.11031-s001.zip › B. Simulation Case 1.docx]

| **MedEdPORTAL Simulation Case**  **SIMULATION CASE TITLE:** Case 1: Mr. K (Precautions – Airborne; Diagnosis – Cocci)  **AUTHORS:** Erin M. Bonura, MD | |
| --- | --- |
| **PATIENT NAME:** Mr. K  **PATIENT AGE:** 45  **CHIEF COMPLAINT:** Fever, night sweats, and cough  **PHYSICAL SETTING:** Inpatient medicine floor | |
|  | |
| **Brief narrative description of case** | Mr. K is a 45-year-old male who presents with several weeks of cough, fever, and night sweats. He has AIDS, treated with antiretroviral therapy and Bactrim, and he works as a counselor at a jail, where he has been exposed to people with tuberculosis (TB). Additionally, he lived in the Southwestern U.S. for many years.  Learner goals:   - Identify the need for airborne precautions based on the concern for pulmonary TB - Don an N95 mask prior to entering the room - Take a subjective history and develop a differential diagnosis for an immunocompromised patient with respiratory symptoms - Correctly identify Coccidioides in a tissue sample - Describe the process of ruling out tuberculosis |
| **Primary Learning Objectives** | 1. Identify the appropriate personal protective equipment (PPE) necessary to examine this patient 2. Demonstrate appropriate donning and doffing techniques for an N95 mask 3. List the differential diagnosis for AIDS patients with respiratory symptoms |
| **Critical Actions** | Based on the patient’s symptoms and risk factors, recognize that pulmonary TB is the leading differential diagnosis.  Recognize that suspected TB requires airborne precautions, then don an N95 mask before entering the patient’s room.  Collect a subjective history that informs the differential diagnosis, including soliciting information about duration of symptoms, quality and frequency of cough, presence of fevers and night sweats while in the hospital.  Access the patient’s transbronchial biopsy results (an unread pathology slide) in the electronic medical record.  Recognize Coccidioides from the pathology image and inform the patient of the diagnosis.  Enter airborne precautions orders in the electronic medical record.  Correctly doff N95 mask –should be removed after leaving the room with the door closed, without touching the front of the mask, and performing hand hygiene after removal. |
| **Learner Preparation or Prework** | Learners complete prework (see Appendix A), which is a power point presentation that describes types of transmission-based precautions, hand hygiene, and correct donning and doffing techniques. |

| Initial Presentation | | | |
| --- | --- | --- | --- |
| **Initial vital signs** | Vital signs are not provided. | | |
| **Overall Setting and Appearance** | Setting: inpatient medical floor. The mannequin is in a hospital bed, in no acute distress. | | |
| **Confederates (e.g., standardized participants) and their roles in the room at case start** | The facilitator (an infectious diseases fellow or physician) speaks through a microphone in the control room and acts as the patient. The patient will answer subjective history questions when asked by the students. Facilitators are not provided with a script; they answer questions as they feel is appropriate based on the clinical syndrome. | | |
| **HPI** | The HPI is provided on the door card, which the students read prior to interacting with the simulated patient:  You are just coming on to the medicine service and are rounding on your first patient. Mr. K is a 45-year-old man with a history of HIV on highly active anti-retroviral therapy (HAART) (CD4 100, viral load 120) and Bactrim who comes in with complaints of fevers, night sweats and cough for “weeks.” He said he started feeling poorly a couple weeks ago and it progressively got worse prompting him to come in. He was seen in the ER and CXR showed a right upper lobe pneumonia. He was started on ceftriaxone and azithromycin then admitted to your team. On the H&P you reviewed, it states that he was born and raised in Arizona and moved to Southern California 15 years ago before moving up to Oregon last year. He denies any history of TB, though he has known many cases of TB having worked full time as a counselor in homeless shelters and jails. Please get a subjective history from the patient and answer his questions to the best of your ability – no need to examine. Please also enter any infection control or diagnostic orders as you see fit.  Subjective:  Facilitators are instructed to answer additional questions in a way they feel is consistent with the diagnosis of pulmonary coccidioidomycosis. We have included a list of the most common questions and recommended answers below:   - Are you feeling any better today since admission? No, I am still coughing and feel like I have a fever. - Night sweats? None last night, but it’s happened a few times over the past few weeks. On those nights, I have been soaking through pajamas and sheets and need to change them. - Any hemoptysis? No, I have not noticed any blood in my cough. - Weight loss? No, my weight has been stable. - GI symptoms? No nausea, vomiting, or diarrhea. My appetite is normal. - Do you take your HIV medications regularly? I have a hard time remembering, and I probably miss them about half the time. | | |
| **Past Medical/Surgical History** | **Medications** | **Allergies** | **Family History** |
| HIV with CD4 of 100 and viral load of 120 (on door card) | Provided on door card:  Highly active antiretroviral therapy (HAART)  Bactrim | No known allergies (if asked) | Students are not expected to ask – facilitator can provide a history if asked |
| **Physical Examination –** Students are not asked to examine this mannequin. Generally, the patient is alert, in no distress, and speaking in full sentences. | | | |

| Instructor Notes - Changes and CASE Branch Points | | |
| --- | --- | --- |
| **Intervention / Time point** | **Change in Case** | **Additional Information** |
| Start of the case | Students will enter the room and begin to collect a subjective history |  |
| 8-10 minutes: Patient will ask the team about diagnosis if students have not already informed him. | Students will log in to electronic medical record, access the patient’s chart, and find the pathology specimen under the media tab. They are provided with a tissue sample with classic *Coccidiodes* spherules and should be able to recognize the image and state the diagnosis. | A sample image:  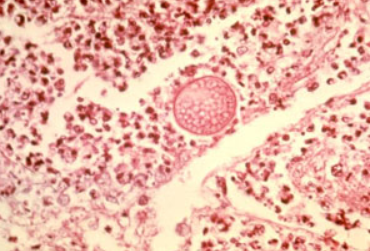  Image by Centers for Disease Control and Prevention, retrieved from <https://www.cdc.gov/fungal/diseases/coccidioidomycosis/health-professionals.html>on 4/6/20. Image is in the public domain.  “ |
| 15 minutes | Facilitator cues students to conclude patient interaction if not already completed and begins debriefing with the students. Debriefing content is listed under “ideal scenario flow.” |  |

**Ideal Scenario Flow**

The students read the door card (under “HPI” above) and realize that the patient may have tuberculosis. They then identify that airborne precautions are required and don N95 masks from the personal protective equipment (PPE) cart, perform hand hygiene, and enter the patient’s room. The team of students then completes a focused subjective history. Once completed, they open the patient’s electronic medical record and access the pathology image under the “media” tab. There is no report attached to the image; they are expected to recognize the classic features of *Coccidiodes.* Students should also recognize that, while TB is less likely given an alternative diagnosis, it is not yet “ruled out.” (If groups do not volunteer this information to the patient, facilitators will frequently ask, “Does this mean I do not have TB?”) Students then place airborne isolation orders in the electronic medical records; they can discuss a treatment plan with the patient, but are not expected to do so. Students should conclude the encounter after about 15 minutes. Finally, they should remove their N95 masks after leaving the patient rooms and closing the door, without touching the front of the mask, and perform hand hygiene after the masks are removed.

After the scenario is completed, students spend 15 minutes debriefing with the facilitator. We provide the following instructions for the debriefing session in the facilitator guide:

- PPE choice: The patient has AIDS and given his exposure history is at risk of TB. His CXR shows upper lobe disease which until proven otherwise, must be ruled out for TB. You should go in with airborne precautions. Airborne only requires a PAPR or N95 mask. IF you have sent a respiratory panel or are concerned about an infection that is spread by fomites, then you can put on a gown – just do not put on a gown for every TB patient as it is not required for Tb given transmission of TB is not by fomite.
- Usually there is an ante room
- Review Don/doff techniques if questions (see pre-work)
- N95 should be snug and not fog up your glasses.
- They likely do not completely understand the difference between and AFB smear and an AFB culture – the process of ruling out active TB.
- In active disease, to diagnose TB, the patient must have 3 sputum samples sent for AFB with 2 of the 3 sent as well for PCR. The sputum should be “induced sputum” performed by respiratory therapy. You can send the lab a sample every 8 hours as long as one is an early morning sample.
- One bronchoalveolar lavage (BAL) negative for AFB does not rule it out but given the biopsy, this is most likely coccidioidomycosis given the spherule seen here. This is a dimorphic fungus (like Histoplasma, blastomyces, and paracoccidioides). The patient lived in the southwest where this is endemic and HIV patients are at risk for endemic mycosis reactivation (progression).
- They may want to send a quantiferon. Note a quantiferon gold and a PPD skin test are SCREENING tests used to screen patients for latent infection. These are NOT to be used in anyone you are concerned may have active disease. It is helpful to explain latent tuberculosis (LTBI) vs active TB
- Pictures are typically in the media tab or can be embedded in progress notes.
- Feel free to talk more about the public health aspects of diagnosis and treatment of TB.

**Anticipated Management Mistakes**

1. Incorrect PPE choice: Almost all groups correctly identify the need for N95 masks, but students will often don disposable gowns and gloves as well, which is not necessary. TB is transmitted by aerosolized droplets and not fomites, so contact precautions are not required.
2. Incorrect donning/doffing techniques: Many students will touch the front of their masks and/or their faces while doffing; we emphasize that they should be handled by the straps only if possible. Many will remove the mask in the patient’s room, as we do not have an anteroom available. We remind students that on the floor, masks should always be removed after leaving the patient’s room and the door is closed.
3. Confusion about terminology: When asked, many students will say the patient needs “respiratory” precautions. We ensure that students understand there are two types of masking procedures – airborne precautions is for infections that spread by small-diameter aerosolized droplets that can remain suspended in the environment for a prolonged period. Droplet precautions are used for larger respiratory droplets that are suspended in the air for a shorter period; surgical masks provide sufficient protection.
4. Misidentification/inability to identify the organism on pathology as *Coccidiodes.* The image displayed is characteristic, with spherules within a granuloma.
5. Students may not recognize that, though there is a presumptive diagnosis of coccidioidomycosis, there is still concern for TB, and that airborne precautions should be maintained until the patient has been “ruled out.”
