# Supplementary material for: Infectious Diseases Simulation for Medical Students: Experiential Instruction on Personal Protective Equipment
Source: MedEdPORTAL. 2020 Nov 24;16:11031. doi: 10.15766/mep_2374-8265.11031 (PMC7703477; doi:10.15766/mep_2374-8265.11031)
Supplement: Supplementary file 1 — Prework Slides.pptxSimulation Case 1.docxSimulation Case 2.docxSimulation Case 3.docxExam Questions.docxEvaluation Questions.docx [file mep_2374-8265.11031-s001.zip › C. Simulation Case 2.docx]

| **Appendix C: Case 2 – Mr. P**  **SIMULATION CASE TITLE: Case 2: Mr. P (Precautions – contact plus; Diagnosis – *C. difficile* colitis)**  **AUTHORS: Erin M. Bonura, MD** | |
| --- | --- |
| **PATIENT NAME: Mr. P**  **PATIENT AGE: 25**  **CHIEF COMPLAINT: Diarrhea**  **PHYSICAL SETTING: Inpatient floor – family medicine service** | |
|  | |
| **Brief narrative description of case** | The patient is a 25-year-old male who presents with profuse diarrhea and abdominal pain several days after receiving a course of amoxicillin for sore throat. *C. difficile* test is positive.  Overall learner goals:   - Recognize that new-onset profuse diarrhea and abdominal pain following antibiotic use is suspicious for *C. difficile.* - Recognize that suspected *C. difficile* requires contact plus transmission based precautions, which require donning a gown and gloves and hand hygiene with soap and water after leaving the patient’s room. - Understand that oral vancomycin is the first-line treatment for *C. difficile* colitis. Metronidazole is not considered first line therapy. |
| **Primary Learning Objectives** | 1. Identify the appropriate personal protective equipment (PPE) necessary for examining this patient 2. Within the electronic medical record, place an order for infection control precautions and find lab data 3. State at least 2 treatment options for *C. difficile* infection |
| **Critical Actions** | Recognize *C. difficile* colitis as most likely diagnosis given the patient’s recent antibiotic exposure and symptoms of diarrhea and abdominal pain.  Based on suspicion for *C. difficile*, don disposable gown and gloves before seeing the patient.  Access positive *C. difficile* testing in the patient’s electronic medical record.  Recognize that two front-line treatments for *C. difficile* are oral vancomycin and fidaxomicin.  Explain the diagnosis and treatment plan to the patient.  Place contact plus precautions order in the EMR.  Wash hands with soap and water – not hand sanitizer – after removing gown and gloves and before exiting the patient’s room. |
| **Learner Preparation or Prework** | Learners complete pre-work (see Appendix A), which is a PowerPoint presentation that provides a review of transmission-based precautions, hand hygiene, and donning and doffing techniques. |

| Initial Presentation | | | |
| --- | --- | --- | --- |
| **Initial vital signs** | Vital signs are not provided for this case. | | |
| **Overall Setting and Appearance** | The mannequin is in a hospital bed in no distress. | | |
| **Confederates (e.g., standardized participants) and their roles in the room at case start** | The facilitator speaks through a microphone as the patient, who remains in stable condition and in the hospital bed throughout the encounter. | | |
| **HPI** | HPI is provided to the students on the door card:  You are on the Family Medicine service seeing your new admissions from overnight. Mr. P is a 25 y/o male admitted through the ED overnight with severe diarrhea. Pt states he was given amoxicillin for a sore throat but 3 days into therapy he developed profuse diarrhea and abdominal pain. In the ED a c difficile was sent and is pending. Please go in the room and check labs for the C. diff result, inform the patient what you would do for management, and place infection control or diagnostic orders if needed.  Students are not expected to obtain additional history or subjective history for this case. Many groups will ask the standardized patient how they are feeling. Frequently asked questions and sample answers:   - Are you feeling any better today? No, I am still having diarrhea and abdominal pain - How many bowel movements have you had so far today? 4 this morning all very loose - Are you having abdominal pain? Yes, lots of cramping pain, and even worse with bowel movements - Any blood in your stool? No, none that I have noticed - Any nausea or vomiting? No, no nausea or vomiting, though I am not feeling hungry - Any fevers? No   Additional information provided to facilitators: the patient has a metronidazole allergy. Facilitators are asked to volunteer this information spontaneously, even if the students do not ask about allergies. The reaction is rash, which occurred many years ago. | | |
| **Past Medical/Surgical History** | **Medications** | **Allergies** | **Family History** |
| Not provided (students are not cued to ask) | Recent amoxicillin use; otherwise, not provided | Metronidazole – facilitator will volunteer if students do not ask | Not provided (students are not cued to ask) |
| **Physical Examination**  Students are not expected to perform a physical examination for this case. The patient is lying comfortably in bed and in no acute distress. | | | |

| Instructor Notes - Changes and CASE Branch Points | | |
| --- | --- | --- |
| **Intervention / Time point** | **Change in Case** | **Additional Information** |
| Start of case | Students recognize need for contact plus precautions, don gown and gloves, and enter patient’s room |  |
| 2-3 minutes | Students access patient’s electronic chart and locate positive *C. difficile* result |  |
| 3-4 minutes | When students counsel patient about *C. difficile* diagnosis, patient reveals metronidazole allergy and asks about the treatment plan |  |
| 4-10 minutes | Students explain treatment plan to patient and place EMR orders for contact plus precautions |  |
| 15 minutes | Facilitator prompts students to conclude patient encounter if not already done, then begins the debriefing session. |  |

**Ideal Scenario Flow**

Students will read the door card and recognize that *C. difficile* colitis is the most likely diagnosis based on the patient’s symptoms and recent antibiotic use. As a result, they should don a disposable gown and gloves, then enter the patient’s room. Though students are not instructed to collect a medical or subjective history, many students will ask how the patient is doing; frequently asked questions and answers are outlined above. Students should access the patient’s electronic chart and look for *C. difficile* test results in the “lab” section. They will find a positive test result. After that, the patient will volunteer (either prompted or unprompted) that he has a metronidazole allergy. Students will explain to the patient that he has *C. difficile* colitis, likely related to his previous course of amoxicillin, and that he requires treatment. They will explain that first line treatment is oral vancomycin, with fidaxomicin as an alternative. They will place an order in EMR for contact plus precautions, and possibly for oral vancomycin. After they finish their conversation with the patient, they will doff gown and gloves and then perform hand hygiene by washing hands with soap and water. The patient encounter should be completed within 15 minutes.

After completing the case, students will debrief with the facilitator. We suggest the following teaching points in the facilitator guide:

- New Name – *Clostridi****oides*** *difficile* PPE for an ordered or positive c difficile test is contact plus. The difference between this and contact is that one must wash hands with soap and water NOT use the alcohol based washes.

Given the IDSA guidelines from 2018, vancomycin PO is now the first line with fidaxomicin as an alternative. Metronidazole is **not** to be used unless the patient cannot take vancomycin or fidaxomicin.

- Also note that though the guidelines state alcohol based washes are “ok” for endemic environments, soap and water is preferred and we want the students to solidify this (soap and water) in their frameworks for PPE and Infection Control.
- Please show your other resources, including IDSA guidelines.
- May discuss clinical or epidemiologic aspects of C*. difficile* as they come up.
  - Risk factors for *C. difficile*
  - Testing algorithm – PCR versus toxin
  - Stewardship of testing – definition of diarrhea and when it is appropriate to test, frequency of testing, possibility of colonization versus true infection

**Anticipated Management Mistakes**

- PPE: students usually recognize that gown and gloves are required. They may attempt hand hygiene with hand sanitizer rather than soap and water.
- Students may not recognize the difference between contact precautions, which require gown and gloves, and contact plus precautions, which also require handwashing with soap and water as well as additional environmental cleaning procedures. Contact plus precautions are generally used for spore-forming organisms.
- Many students are not aware that metronidazole is no longer considered first-line therapy for *C. difficile* infection due to multiple studies showing higher clinical and microbiologic success rates with vancomycin.
- Most can name oral vancomycin as an option, but fewer are aware of fidaxomicin as the oral first-line antibiotic therapy, nor are they aware of the primary barrier (cost).
- Though instructed to place orders for contact plus precautions in the door card, many students need prompting to place this order.
- Students will sometimes attempt to repeat a full history and physical exam, and need to be reminded that the focus of the case is on diagnosis and treatment.
