# Supplementary material for: Infectious Diseases Simulation for Medical Students: Experiential Instruction on Personal Protective Equipment
Source: MedEdPORTAL. 2020 Nov 24;16:11031. doi: 10.15766/mep_2374-8265.11031 (PMC7703477; doi:10.15766/mep_2374-8265.11031)
Supplement: Supplementary file 1 — Prework Slides.pptxSimulation Case 1.docxSimulation Case 2.docxSimulation Case 3.docxExam Questions.docxEvaluation Questions.docx [file mep_2374-8265.11031-s001.zip › D. Simulation Case 3.docx]

| **Appendix D: Case 3**  **SIMULATION CASE TITLE: Case 3: Violet (Precautions – contact and droplet; diagnosis – neutropenic fever)**  **AUTHORS: Erin M. Bonura, MD** | |
| --- | --- |
| **PATIENT NAME: Violet**  **PATIENT AGE: 6**  **CHIEF COMPLAINT: Neutropenic fever**  **PHYSICAL SETTING: Pediatric hematology/oncology unit** | |
|  | |
| **Brief narrative description of case** | Violet is a 6-year-old female who underwent bone marrow transplant three weeks ago and remains neutropenic. It is February, and there is an outbreak of influenza on the floor. Yesterday, she spiked a fever. Initial testing, including blood cultures and urinalysis, are negative thus far. Violet reports symptoms (cough, subjective fever) concerning for flu.  Overall learner goals:   - Recognize that new fever in a patient on a floor with a known influenza outbreak is concerning for flu. - Identify that suspected influenza requires both droplet (mask) and contact (gown and gloves) precautions because flu is spread by both droplet and fomites. - Perform a subjective history and review of systems in a patient with neutropenic fever. - Recognize how to test for influenza. |
| **Primary Learning Objectives** | 1. Given a case, identify the appropriate personal protective equipment (PPE) necessary when examining the patient 2. Articulate the difference between transmission-based precautions and protective (neutropenic) precautions |
| **Critical Actions** | 1. Recognize that a patient with a new fever on a hospital floor with a known influenza outbreak is likely to have influenza 2. Understand that influenza is spread by droplets and fomites, and therefore requires both droplet and contact precautions – don gown, gloves, and surgical mask. 3. Ask patient and/or patient’s mother about subjective symptoms consistent with influenza, including cough, shortness of breath, fever, myalgias. Students should also complete a review of systems 4. Auscultate the patient’s lungs and identify bilateral crackles 5. Explain to the patient and her mother that they will be testing for influenza (and possibly other respiratory viruses.) |
| **Learner Preparation or Prework** | Learners complete pre-work (see Appendix A), which is a PowerPoint presentation that provides a review of transmission-based precautions, donning and doffing techniques, and hand hygiene. |

| Initial Presentation | | | |
| --- | --- | --- | --- |
| **Initial vital signs** | No vital signs provided to the students, but patient’s mother (unprompted) will tell the students that Violet’s temperature was 100.9 overnight. | | |
| **Overall Setting and Appearance** | Violet is in bed, in no acute distress. | | |
| **Confederates (e.g., standardized participants) and their roles in the room at case start** | There are 2 mannequins – Violet is a pediatric mannequin in a hospital bed, and her parent is an adult mannequin who is in a chair at bedside. The facilitator speaks in the microphone as both mannequins. Both the patient and her parent will answer questions about symptoms. Facilitators are not provided with a script, but will answer questions as they think is consistent with the clinical syndrome. | | |
| **HPI** | HPI is provided on the door card:  You are on the pediatric hematology-oncology service. Your patient is a 6-year-old girl with a history of a bone marrow transplant 3 weeks ago on February 16^th^ now with continued neutropenia and new fevers. She had blood cultures drawn which are pending and a urinalysis which was negative. You learn that 2 other patients on the floor have been diagnosed with the flu. Please go in and gather a subjective history on the patient and answer her questions. Please listen to her lungs, check her labs, and enter diagnostic orders for further work up as you see fit.  Subjective history:  Students are asked to solicit a subjective history from Violet and her mother. Frequently asked questions and answers are listed below:   - Is Violet having subjective fever? Yes, she is having bouts of shivering and sweats. A nurse took her temperature overnight and told us it was 100.9 - Is she having body aches? Yes, she “hurts all over” - Any nasal congestion? No - Any cough? Yes, she developed a dry cough overnight. It is not productive of any sputum or blood - Any shortness of breath? No, no shortness of breath - Any GI symptoms? Violet is not having nausea, vomiting, or diarrhea. However, her appetite is significantly decreased from usual. - Any dysuria, urinary frequency, or urgency? No, she has had no changes in urination - Any skin rashes? No - Any swelling of her extremities? No - Have any of her visitors been ill? No, only her parents have been to visit and both are well. | | |
| **Past Medical/Surgical History** | **Medications** | **Allergies** | **Family History** |
| BMT 3 weeks ago | None/students are not cued to ask | If students ask, no known drug allergies | Students are not cued to ask – facilitators may provide an answer if asked |
| **Physical Examination** Violet is coughing intermittently but resting comfortably. Students are asked to examine only her lungs. | | | |
| **Lungs** | With hi-fidelity simulation stethoscope, students should auscultate bilateral crackles. | | |

| Instructor Notes - Changes and CASE Branch Points | | |
| --- | --- | --- |
| **Intervention / Time point** | **Change in Case** | **Additional Information** |
| Start of case | Students read the door card and are concerned for influenza. They don gown, gloves, and droplet mask |  |
| Minutes ~1-10 | Students gather subjective history |  |
| Minutes ~1-10 | Students auscultate patient’s lungs with high-fidelity stethoscope and appreciate bilateral crackles |  |
| Minutes 8-10 | If not already addressed by students, Violet’s mother asks about the plan. Students should state that they plan to test her for influenza (+/- other infectious diseases workup) |  |
| Minute 10-15 | Students should place diagnostic orders (influenza PCR) and infection control orders (contact and droplet precautions); facilitators can prompt if not already done. |  |
| 15 minutes | Facilitators should cue students to complete encounter if not already done, then start the debrief session. Students should remove gowns, gloves, and masks and perform hand hygiene. |  |
| 15-20 minutes | After students complete the simulation, facilitators should turn off the lights in the control room and examine the students’ hands for fluorescent powder with a UV flashlight. |  |

**Ideal Scenario Flow**

Prior to the start of the case, the facilitator should spread fluorescent powder, if available, on all high-touch services – bed rails, counters, etc. Power should be spread lightly as possible – ideally, students will not notice it is there, though it is generally visible on most surfaces.

Students should read the door card and recognize that fever in an immunocompromised patient residing on a hospital floor with a known influenza outbreak is concerning for flu. As a result, they should don contact precautions (gown and gloves) and droplet precautions (surgical mask) as flu is spread by both droplet and fomites. They should then enter the patient’s room. Subjective history should focus on influenza-like symptoms, though students should recognize the need for a complete review of systems given the patient’s neutropenic fever. Common questions and answers are outlined in “HPI” above. They should then listen to the patient’s lungs with the hi-fidelity stethoscope and state that the patient has bilateral crackles. After collecting this information, they should discuss a plan with the patient and her parent. Students will frequently discuss ordering other testing (respiratory viral panel, additional blood cultures) or starting antibiotics. These plans can be discussed with the facilitator during the debriefing session; we encourage facilitators to offer a basic overview of neutropenic fever management, though we do not expect students to be prepared to manage this diagnosis without prompting. Students will place diagnostic orders, contact precautions and droplet precautions orders. Facilitators will prompt students to complete the patient interaction around 15 minutes if they have not already done so. The students should remove their masks, gowns, and gloves, then perform hand hygiene. The facilitator opens the debrief session by turning off the lights in the control room and using a UV flashlight to examine the hands and arms of the students for the presence of the fluorescent powder – students have presumably come in contact with the powder while in the room and removing PPE, and may have some powder remaining on their hands in areas that are frequently missed during hand hygiene.

In the facilitator guide, we provide the following teaching points:

- PPE - don contact and respiratory PPE. Though flu is spread by respiratory droplets, it is also spread by fomites - transferring the droplets via contact to mucus membranes (shaking hands, touching eyes.) Look at black light spots on student.
- Note that patient should be on protective precautions. Compare Protective Precautions to the Transmission Based Precautions. These do not mandate gown, gloves, or mask for the provider. PPT pre-work should have a picture of this for the students. Please inform them that if they are ill, they should not be at work (certainly not entering rooms of immunocompromised patients).
- Given the other positive flu diagnoses on the floor, would don prior to entering the room (even though it is not specified on the room yet) and order a flu test
- They can place an order for combination precautions when they order the flu test
- Would discuss cost of flu/RSV PCR versus full respiratory viral panel – if suspicion for influenza is high, as it is here, it may be appropriate/cost saving to start with the cheaper influenza test and proceed to the respiratory viral panel if negative
- May order a CXR as well, though not required given her relative lack of lower respiratory symptoms
- May order a full respiratory viral panel if flu comes back negative.
- Can discuss neutropenic fever work up, empiric treatment if time/or it comes up. Highlights: empiric anti-pseudomonal antibiotics are started immediately for neutropenic fever. Blood cultures are sent as a matter of routine and additional workup depends on patient history and symptoms. Neutropenic fever is considered an emergency/urgent situation and needs to be responded to quickly.

**Anticipated Management Mistakes**

- Students will generally recognize the necessary transmission-based precautions for suspected influenza. However, when asked during the debrief to define protective precautions for neutropenic patients and compare them to transmission-based precautions, many students are under the mistaken impression that they need to wear masks (+/- gowns and gloves) for *all* neutropenic patients. It is important that students leave the debrief session with an understanding of the rationale behind protective precautions and how to implement them, and be example to explain how they compare to transmission-based precautions. Our most commonly used explanation is that transmission-based precautions are to protect yourself from the patient’s infection, and protective precautions are to protect vulnerable patients from *you* and the hospital environment.
- Students will generally recognize the need for influenza testing. Their plans for other infectious diseases workup and antibiotic management tend to vary widely. Neutropenic fever is a complex concept and we do not expect the students to have a strong handle on how to manage it. The debrief sessions should first focus on the differences between protective and transmission-based precautions, and the correct PPE to use for suspected influenza. Then, facilitators can provide a brief overview of neutropenic fever management, but should recognize that this concept is not the key learning point of the case.
- Students often notice the fluorescent powder in the room and will ask about its significance; we generally encourage them to proceed through the case as they normally would.
