# Supplementary material for: Infectious Diseases Simulation for Medical Students: Experiential Instruction on Personal Protective Equipment
Source: MedEdPORTAL. 2020 Nov 24;16:11031. doi: 10.15766/mep_2374-8265.11031 (PMC7703477; doi:10.15766/mep_2374-8265.11031)
Supplement: Supplementary file 1 — Prework Slides.pptxSimulation Case 1.docxSimulation Case 2.docxSimulation Case 3.docxExam Questions.docxEvaluation Questions.docx [file mep_2374-8265.11031-s001.zip › E. Exam Questions.docx]

Simulation Lab Exam Questions

You are given a sign out on a patient with *C. difficile* colitis. What is the difference between the infection precautions for your patient versus a patient with MRSA?

A)      *C. difficile* patients require gown and gloves

B)      MRSA patients require splash guards

C)      *C. difficile* patients require hand washing

D)      MRSA patients require standard precautions

Correct answer: C

You just examined your patient with pulmonary tuberculosis with proper airborne precautions and personal protective equipment. What is the last item of PPE you should remove after exiting the room while standing in the anteroom?

A)      Gloves

B)      Goggles or face shield

C)      Gown

D)      Mask

Correct answer: D

Assuming hand hygiene is performed well, what personal protective equipment must one “don” when working with a patient that has influenza?

A)      Gown, gloves, N95 mask

B)      Gown, gloves, droplet mask

C)      N95 mask and gown

D)      Droplet mask and gloves

Correct answer: B
