# Supplementary material for: Infectious Diseases Simulation for Medical Students: Experiential Instruction on Personal Protective Equipment
Source: MedEdPORTAL. 2020 Nov 24;16:11031. doi: 10.15766/mep_2374-8265.11031 (PMC7703477; doi:10.15766/mep_2374-8265.11031)
Supplement: Supplementary file 1 — Prework Slides.pptxSimulation Case 1.docxSimulation Case 2.docxSimulation Case 3.docxExam Questions.docxEvaluation Questions.docx [file mep_2374-8265.11031-s001.zip › F. Evaluation Questions.docx]

**Appendix F: Student Evaluation Form**

The session was well organized


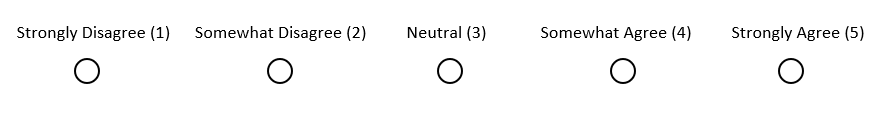


The educational material and resources enhanced my learning


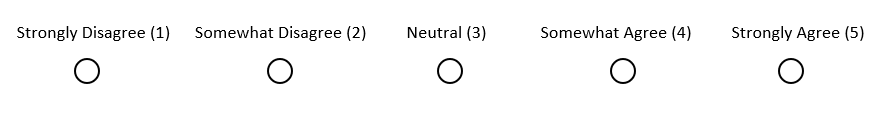


Additional comments and suggestions:
