# Supplementary material for: Infectious Diseases Simulation for Medical Students: Experiential Instruction on Personal Protective Equipment
Source: MedEdPORTAL. 2020 Nov 24;16:11031. doi: 10.15766/mep_2374-8265.11031 (PMC7703477; doi:10.15766/mep_2374-8265.11031)
Supplement: Supplementary file 1 — Prework Slides.pptxSimulation Case 1.docxSimulation Case 2.docxSimulation Case 3.docxExam Questions.docxEvaluation Questions.docx [file mep_2374-8265.11031-s001.zip › _Educational_Summary_Report_11031.pdf]

MedEdPORTAL® The AAMC Journal of Teaching and Learning Resources

Original Publication Open Access

Infectious Diseases Simulation for Medical Students: Experiential Instruction on Personal Protective Equipment

Angela Holly Villamagna, MD*, Erin M. Bonura, MD, MCR

*Corresponding author: villamag@ohsu.edu

Abstract

Introduction: The emergence of COVID-19 highlighted the critical importance of appropriate use of personal protective equipment (PPE) for the safety of patients and health care personnel. However, previously published survey data indicated that formal instruction on the correct utilization of PPE is uncommon in medical school curricula, and there is no published guidance about optimal instruction methods. The infectious disease (ID) simulation lab at Oregon Health & Science University filled this need. **Methods:** Second- through fourth-year medical students participated in the infection intersession, a 2-week didactic session that students were required to enroll in once during their clinical rotations. As part of the course, students completed the ID simulation lab, during which they were presented with common ID syndromes (suspected tuberculosis, *C. difficile* colitis, and neutropenic fever) and asked to select the proper PPE prior to interacting with standardized patients. ID physicians acted as the patients and then conducted feedback sessions, which focused on PPE choice, donning and doffing techniques, and ID diagnosis and management principles. **Results:** More than 500 medical students participated between 2016 and 2020, demonstrating the feasibility of the experience. The average exam scores were above 80%, and the average student evaluation score of the session was 8.9 out of 10, demonstrating acceptability. **Discussion:** The ID simulation lab allowed students to reinforce didactic teaching about PPE, dispel common misconceptions, and receive real-time feedback from ID clinicians. Availability of the lab and facilitators were limiting factors. Future work will focus on better understanding the efficacy of the sessions.

Keywords
Simulation, Infectious Diseases, Infection Control, Personal Protective Equipment, C. difficile, AIDS, Tuberculosis, Influenza

Educational Objectives

By the end of this activity, learners will be able to:

1. Identify the appropriate personal protective equipment necessary for a patient.
2. Differentiate protective precautions from transmission-based precautions.
3. Describe treatment options for C. difficile.
4. List a differential diagnosis for HIV patients with respiratory complaints.
5. Input infection control orders and locate diagnostic data within an electronic medical record.

Citation:
Villamagna AH, Bonura EM. Infectious diseases simulation for medical students: experiential instruction on personal protective equipment. *MedEdPORTAL*. 2020;16:11031.
https://doi.org/10.15766/mep_2374-8265.11031

Introduction

The correct use of personal protective equipment (PPE) is crucial for both infection control and health care worker safety. The recent emergence of novel coronavirus (COVID-19) has prompted important questions about whether medical personnel are adequately trained on PPE selection, donning and doffing techniques, and how to best deliver necessary education. Though practicing physicians make daily decisions about PPE, the available literature suggested that the quantity and quality of education on PPE during medical school is inconsistent. A 2017 survey of Northeastern Ohio medical students demonstrated that 60% did not recall training in the use of PPE.[1] The majority of resident and attending physicians, surveyed as part of the same study, and representing 67 medical schools, also reported that they had not received any formal education on PPE use.[1] Though these surveys may be limited by incomplete recall, the findings nonetheless suggested that many physicians are not receiving formal training on PPE during medical school.

Copyright © 2020 Villamagna and Bonura. This is an open-access publication distributed under the terms of the Creative Commons Attribution license.

More broadly, it is well established that health care worker compliance with recommended PPE is not consistent and that PPE is often donned and doffed incorrectly when worn; surveys revealed gaps in understanding and inconsistent implementation.[2,3] In the absence of formal teaching, medical students learn about PPE on the job and by example and are therefore frequently modeling suboptimal techniques.

Though gaps in medical student PPE curriculum have been identified, the optimal method of delivering this content has yet to be examined, and to our knowledge there are no published curricula. At Oregon Health & Science University (OHSU), students were introduced to the correct use of PPE through small-group case vignettes during their preclinical microbiology block. The same concepts were taught again via PowerPoint presentation during the Transition to Clinical Medicine course, which was held immediately before students started clinical clerkships. During their clinical rotations we utilized the simulation lab experience to directly observe students' practices and offered feedback on their choices and techniques, which allowed them to solidify what they learned in the previous didactic sessions and to correct misconceptions they may have encountered in hospitals and clinics. Understanding that decisions about PPE are made in real time at the bedside, and often before a patient's diagnosis and management plan are established, the simulation lab was an appropriate setting for teaching as it best captured the decision points clinicians face on the wards. The simulated cases first asked students to make choices about which PPE to don based on an introductory patient vignette. The remainder of the experience allowed students to work through differential diagnoses, diagnostic testing, and treatment of common infectious diseases (IDs).

Methods

Development

The simulation lab experience was developed for medical students in their clinical clerkships at OHSU. It was scheduled during the infection intersession, a 2-week didactic and experiential course covering ID concepts that each student must complete once during their clinical rotations; enrolled students were in their second, third, or fourth years of medical school. The intersession was held three times a year, and approximately 60 students were enrolled in each session.

The simulation lab session ran for 4 hours total, and each group of students spent 1-1.5 hours rotating through cases. We ran three cases simultaneously.

Prior to the session, students completed prework, independently reviewing a 26-slide presentation covering basics of infection control and PPE. They were provided with three questions to answer for themselves to guide their review (Appendix A).

Equipment/Environment

The session took place at our medical school's simulation lab. Recommended equipment included:

- Three adult mannequins and one pediatric mannequin.
- If available, programmable stethoscope for case 3, a patient with bilateral crackles on respiratory exam, or a high-fidelity mannequin capable of producing lung sounds.
- Hallway camera with video feed viewable in the control room—allowed the facilitator to observe donning of PPE (if not available, the facilitator could directly observe this process).
- Information cards posted outside the patient room describing the case scenario.
- PPE cart outside each room with gowns, gloves, surgical masks, and N95 masks.
- Computer on wheels with electronic medical record (EMR) access and patient charts built in.
- Glo-Germ or other fluorescent powder (optional for case 3).
- Ultraviolet flashlight (optional for case 3).
- Visual stimuli:
  - Representative chest X-ray for a patient with pulmonary coccidioidomycosis.
  - Representative pathology image of a bronchoalveolar lavage (BAL) sample with spherules of coccidioidomycosis (Appendix B).

Personnel

Medical students participated in small groups. There was a facilitator for each room—in most simulation sessions, there were a total of six rooms running simultaneously, so there were two facilitators per case, each working with different groups of students. Facilitators were ID fellows and attending physicians, though infection control personnel with clinical backgrounds or hospitalists well versed in PPE could have potentially acted as facilitators. Additionally, multiple simulation lab program technicians were available to escort groups of students between stations, assist with setup, and troubleshoot any technical difficulties.

Implementation

Groups of two to four students interacted with the simulated patient and then participated in the feedback session. We preferred to keep groups at four students or fewer to give all

Copyright © 2020 Villamagna and Bonura. This is an open-access publication distributed under the terms of the Creative Commons Attribution license.

students an opportunity to converse with the simulated patient and contribute to decision making. Students were allotted 30-40 minutes per case depending on availability of rooms and facilitators; they spent 15-20 minutes with the simulation patient, followed by 15-20 minutes receiving feedback from the facilitator. All groups completed all three cases.

When students arrived at the simulated patient's room, they were provided with an informational card on the door that introduced them to the patient's chief complaint and symptoms (see history of present illness in Appendices B-D). The card prompted them to don the PPE they thought was correct from a cart outside the room before entering. The facilitator sat in a control room behind a one-way mirror, allowing them to observe the patient interaction. Additionally, there were cameras in the hallway that provided a video feed to the facilitator, allowing them to observe the PPE donning process. After the students donned PPE, they entered the patient's room and completed the tasks assigned to them on the instruction card. The facilitator spoke into a microphone, acting as the patient. The facilitator was provided with additional information about the patient on their handouts (see subjective history in Appendices B-D). Facilitators were instructed to answer students' questions in a manner consistent with the clinical syndrome. However, to support non-ID clinicians in facilitating the activity, we have compiled the most frequently asked questions and scripted answers in Appendices B-D. In-room tasks varied by case. Briefly:

- Case 1 (Appendix B) was an adult patient with AIDS who presented with cough, fever, night sweats, and a social history concerning for both tuberculosis (TB) and coccidioides exposures. Students were expected to recognize the need for airborne precautions. They were instructed to obtain a history from the patient and check the EMR for the results of his BAL, where they found a pathology specimen diagnostic of coccidioidomycosis. The feedback session focused on correct utilization of airborne precautions and the processes for diagnosing tuberculosis and coccidioidomycosis.
- Case 2 (Appendix C) was a 25-year-old patient with recent antibiotic exposure admitted for diarrhea, which prompted concern for C. difficile. Students were expected to utilize contact plus precautions (gown, gloves, and hand hygiene with soap and water). They were instructed to access his C. difficile result in the EMR and inform the patient of the diagnosis and their treatment plan. The feedback session focused on contact plus precautions and guidelines for treatment of C. difficile.
- Case 3 (Appendix D) was a pediatric bone marrow transplant patient with neutropenic fever. There was an influenza outbreak on the hospital floor. Students were asked to obtain a history, examine the patient's lungs with a high-fidelity stethoscope that simulated crackles, and offer a plan to the patient's family. The students were expected to utilize contact and droplet precautions for suspected influenza. The feedback session focused on contrasting protective (neutropenic) precautions with transmission-based precautions and diagnosis of influenza.

Feedback

Medical student groups then received feedback from the facilitator in the control room immediately after the simulation session. Facilitators were provided with a list of key points to discuss with students for each case (see ideal scenario flow in Appendices B-D). Facilitators were instructed to open the session by asking students to explain their PPE choice, then offer feedback on the correct PPE for the scenario as well as donning and doffing procedures. As time allowed, the discussion proceeded into diagnostic and treatment considerations relevant to the patient case. Facilitators were provided with detailed teaching points on the handout, but could add additional content based on students' questions or areas of interest.

High Enrollment Implementation

Increased enrollment occasionally required larger groups of students (up to 6), where each group of students participated in only two simulated cases. This scenario is outlined in the sample schedule (Figure). Additionally, when teaching groups of more than four students, we have asked one to two students to act as observers during each case. These students sat in the control room with the facilitator and watched the rest of the group interact with the simulated patient. Different students acted as observers during each case. All students participated in the debrief session.

Assessment

Students were not formally assessed on their performance during the simulation session, as we designed the session as a formative, low-stakes experience. However, after the session students were asked to assign a score of 1 (*strongly disagree*) to 5 (*strongly agree*) for two questions: "The session was well organized," and, "The educational material and resources enhanced my learning" (Appendix F). The form also provided space for comments. In addition, PPE and infection control principles were tested in the end-of-course multiple-choice exam. There were three questions based on the simulation

Copyright © 2020 Villamagna and Bonura. This is an open-access publication distributed under the terms of the Creative Commons Attribution license.

Time (minutes)	Case 1 Facilitator Room 1	Case 2 Facilitator Room 2	Case 3 Facilitator Room 3	Case 1 Facilitator Room 4	Case 2 Facilitator Room 5	Case 3 Facilitator Room 6
0-40	Group 1	Group 2	Group 3	Group 4	Group 5	Group 6
40-80	Group 3	Group 1	Group 2	Group 6	Group 4	Group 5
80-120	Group 7	Group 8	Group 9	Group 10	Group 11	Group 12
120-160	Group 9	Group 7	Group 8	Group 12	Group 10	Group 11
160-200	Group 13	Group 14	Group 15	Group 16	Group 17	Group 18
200-240	Group 15	Group 13	Group 14	Group 18	Group 16	Group 17

Figure. Sample high enrollment schedule for the infectious diseases simulation lab, including 18 groups of six students. Learners complete two cases instead of three.

lab experience (Appendix E), which covered multiple concepts introduced during the session:

- Question 1: C. *difficile* and need for hand hygiene with soap and water.
- Question 2: TB and doffing procedures for airborne isolation.
- Question 3: influenza and required PPE.

Results

The simulation lab experience was introduced in March 2016. Since then, we have held the session 12 times for more than 500 total students. Of students, 401 submitted evaluations. After summing response to the two 5-point Likert questions, the average of all student evaluations was 8.9 out of 10.

In positive evaluations, students expressed appreciation for the opportunity to practice skills outside the hospital in a safe and low-pressure environment, highlighted the utility of the simulation lab experience in consolidating concepts introduced in lecture, and most commonly, expressed appreciation for having access to ID clinicians for teaching and feedback in a small-group setting, as expressed by the representative comments:

- “I appreciate that the stakes are low in this situation, and we are given the opportunity to make mistakes and learn from them in a supportive space. We will have plenty of opportunities to learn in a high-stakes environment, so I really like getting to do this first.”
- “Dispelled a few misconceptions I had.”
- “It was good practice to decide what kind of PPE is required for patients with varying infections. The debrief following the cases was a great educational moment.”
- “It was great to practice using PPE correctly. Much more helpful than just being lectured on the material.”

The constructive feedback requested more clarity on our expectations of the students and the goals of the simulation lab prior to starting the session. Students also suggested that facilitators ensure they leave enough time for a thorough feedback session. Finally, student reviewers commented that they preferred to focus on interacting with the simulated patients and facilitators rather than spending time placing orders, which they felt they had sufficient exposure to in other settings:

- “I wish we had a little more of an introduction before we stepped in. I was still grasping the ‘rules’ of what I was allowed to do for the first and second simulations.”
- “I have practiced these PPE things all the time in the hospital when I see patients. The in-class lecture on what PPE is appropriate and when was sufficient, I didn’t need to actually practice it.”
- “My one point of feedback is I think it is way more valuable to participate in the room than observe (although there is some value in observing as well). I know the numbers of students were very large but I would recommend having everyone participate in every case.”
- “As always, I wish that we had more time to discuss with the attendings after the simulation was over. Sometimes our simulations went rather long so maybe a time warning to get students out of the exam room in time to have enough discussion time would also be helpful for next time.”

Of the students who participated, 511 have completed the associated exam questions and have performed well on them:

- Question 1: C. *difficile* and need for hand hygiene with soap and water, 89% correct.
- Question 2: TB and doffing procedures for airborne isolation, 87% correct.
- Question 3: influenza and required PPE, 82% correct.

Discussion

Though appropriate use of PPE is a vital skill for physicians in all specialties, there is a dearth of published curricula to educate medical students about these concepts. Our simulation lab curriculum was created to address the need for experiential teaching in PPE.

Copyright © 2020 Villamagna and Bonura. This is an open-access publication distributed under the terms of the Creative Commons Attribution license.

Strengths

Evaluations for the simulation experience were generally positive and affirmed that the session was acceptable to students. Though students received instruction on appropriate PPE use in preclinical didactic sessions and small-group case studies, and again in their transition to clinical experiences course, decisions about PPE are made in the moment, during patient care. The strength of the simulation lab curriculum was the use of a constructivist approach to learning that mimicked the students' experiences in patient care. They were asked to recall and make meaning of what they were taught in the classroom to make decisions during a clinical scenario. The sessions were facilitated by ID faculty and fellows well versed in PPE; their role was to guide the learners through the choices they made, help them identify and correct assumptions and misconceptions, and deepen their understanding of decisions they make daily. The ultimate goal was for the students to reflect more deeply on their previous perspectives and behaviors surrounding PPE, identify errors, and develop a new framework for PPE selection and application that they could apply on the wards.[4] Anecdotally, students commented that the simulation lab allowed them to better understand practices they have observed in the hospital, and facilitators have noted that they were consistently correcting misconceptions about PPE, which may have otherwise gone unnoticed and unaddressed. We successfully completed 12 simulation lab sessions over the past 4 years, which demonstrated the feasibility of the simulation experience.

Limitations

Interpretation of our student evaluation was limited by a relative lack of free text comments, which are useful in evaluating the students' interpretation of the experience. Though greater than 80% of students answered the associated exam questions correctly, it was unclear if results would be similar if the content were delivered by a more traditional lecture format, and either short answer questions or a practical exam may have allowed us to better assess the students' level of understanding.

One of the primary barriers to successful implementation was simulation lab access: at OHSU, the lab was utilized by multiple professional schools and needed to be scheduled more than a year in advance. Recruiting enough facilitators was also challenging, as the session required a 4-hour time commitment.

Though we preferred to conduct this activity in groups of two, so that each student was heavily involved in standardized patient interaction and decision making, the flexibility of the session has allowed us to accommodate larger groups of up to six students. The amount of time scheduled for each case can be modified to allow for more groups of students to rotate, though we have found that a minimum of 30 minutes per case is necessary to allow adequate time for simulated patient interaction and facilitator feedback. Decreasing the size of the group interacting with the standardized patient allowed the students to be more involved in the cases they participated in; given that experiential learning is the central benefit to this session, we felt that it was important to structure the sessions such that each student was able to make real-time decisions and interact with the simulated patients.

Future Work

Our future work includes developing a fourth simulation lab case that covers standard precautions and lower extremity foot wound management. In response to the need for virtual and remote learning during COVID-19, we are planning a mixed-methods effectiveness study during the next simulation lab session comparing in-person simulation to virtual PPE simulation using the same cases. A group of students will complete an independent study session in lieu of attending the simulation lab; they will receive the same vignettes in a slide deck, access the same patient charts in the EMR, and submit answers to questions electronically; then they will receive an answer key they will be expected to review independently. All students will complete short answer essay exam questions on PPE; responses of students who participated in the simulation lab will be compared to responses of those who did not. Student evaluations will also be compared. We hypothesize that the in-person simulation lab will be more acceptable and effective, but recognize the benefit of having a feasible virtual alternative due to the ongoing COVID-19 pandemic.

Appendices

A. Prework Slides.pptx
B. Simulation Case 1.docx
C. Simulation Case 2.docx
D. Simulation Case 3.docx
E. Exam Questions.docx
F. Evaluation Questions.docx

All appendices are peer reviewed as integral parts of the Original Publication.

Copyright © 2020 Villamagna and Bonura. This is an open-access publication distributed under the terms of the Creative Commons Attribution license.

Angela Holly Villamagna, MD: Instructor, Division of Infectious Diseases, Oregon Health & Science University; ORCID: https://orcid.org/0000-0001-9247-2336

Erin M. Bonura, MD, MCR: Associate Professor of Medicine, Division of Infectious Diseases, Oregon Health & Science University

Disclosures
None to report.

Funding/Support
None to report.

Ethical Approval
Reported as not applicable.

References

1. John A, Tomas ME, Hari A, Wilson BM, Donskey CJ. Do medical students receive training in correct use of personal protective equipment? *Med Educ Online*. 2017;22(1):1264125. https://doi.org/10.1080/10872981.2017.1264125

2. Harrod M, Weston LE, Gregory L, et al. A qualitative study of factors affecting personal protective equipment use among health care personnel. *Am J Infect Control*. 2020;48(4):410-415. https://doi.org/10.1016/j.ajic.2019.08.031

3. John A, Tomas ME, Cadnum JL, et al. Are health care personnel trained in correct use of personal protective equipment? *Am J Infect Control*. 2016;44(7):840-842. https://doi.org/10.1016/j.ajic.2016.03.031

4. Torre DM, Daley BJ, Sebastian JL, Elnicki DM. Overview of current learning theories for medical educators. *Am J Med*. 2006;119(10):903-907. https://doi.org/10.1016/j.amjmed.2006.06.037

Received: March 12, 2020
Accepted: July 15, 2020
Published: November 24, 2020

Copyright © 2020 Villamagna and Bonura. This is an open-access publication distributed under the terms of the Creative Commons Attribution license.
